# Culturable Bacterial Endophytes of Wild White Poplar (*Populus alba* L.) Roots: A First Insight into Their Plant Growth-Stimulating and Bioaugmentation Potential

**DOI:** 10.3390/biology12121519

**Published:** 2023-12-12

**Authors:** Natalya S. Gladysh, Alina S. Bogdanova, Maxim A. Kovalev, George S. Krasnov, Vsevolod V. Volodin, Anastasia I. Shuvalova, Nikita V. Ivanov, Mikhail I. Popchenko, Aleksandra D. Samoilova, Aleksandra N. Polyakova, Alexey A. Dmitriev, Nataliya V. Melnikova, Dmitry S. Karpov, Nadezhda L. Bolsheva, Maria S. Fedorova, Anna V. Kudryavtseva

**Affiliations:** 1 Engelhardt Institute of Molecular Biology, Russian Academy of Sciences, Vavilov Str., 32, 119991 Moscow, Russia; natalyagladish@gmail.com (N.S.G.); alina.bogdashka@yandex.ru (A.S.B.); kovalev_maksim_2002@mail.ru (M.A.K.); gskrasnov@mail.ru (G.S.K.); vsevolodvolodin@yandex.ru (V.V.V.); anastasiashuvalova777@gmail.com (A.I.S.); revillnik@mail.ru (N.V.I.); popchenko_m@inbox.ru (M.I.P.); alex_245@mail.ru (A.A.D.); mnv-4529264@yandex.ru (N.V.M.); aleom@yandex.ru (D.S.K.); nlbolsheva@mail.ru (N.L.B.); fedorowams@yandex.ru (M.S.F.); 2 Institute of Agrobiotechnology, Russian State Agrarian University—Moscow Timiryazev Agricultural Academy, 127434 Moscow, Russia; 3 Center for Precision Genome Editing and Genetic Technologies for Biomedicine, Engelhardt Institute of Molecular Biology, Russian Academy of Sciences, Vavilov Str., 32, 119991 Moscow, Russia; 4 Institute of Geography, Russian Academy of Sciences, Staromonetny Pereulok, 29/4, 119017 Moscow, Russia; 5 Faculty of Soil Science, Lomonosov Moscow State University, Leninskie Gory, 1/12, 119234 Moscow, Russia; ribaluna2003@gmail.com (A.D.S.); polyakovaan@my.msu.ru (A.N.P.)

**Keywords:** white poplar, bacterial endophytes, bioaugmentation, *Pseudomonas*, *Kocuria*

## Abstract

**Simple Summary:**

The white poplar tree has great potential in greening cities and protecting the environment from pollution. However, planting poplars on contaminated soils can lead to suppression of poplar growth. To mitigate this problem, endophytes that stimulate plant growth and are able to degrade pollutants can be used. We studied the genomes of 14 different bacteria that live inside the roots of the white poplar tree and found that these bacteria have genes that help the tree grow and resist soil pollution. They contain genes that help the tree utilize important nutrients, produce beneficial chemicals, and get rid of harmful substances. The bacteria we studied are new strains of known bacterial species. The most promising are strains from the genera *Pseudomonas* and *Kocuria*. We believe that these endophytic bacteria can be used together with white poplar to better protect the environment.

**Abstract:**

The white poplar (*Populus alba* L.) has good potential for a green economy and phytoremediation. Bioaugmentation using endophytic bacteria can be considered as a safe strategy to increase poplar productivity and its resistance to toxic urban conditions. The aim of our work was to find the most promising strains of bacterial endophytes to enhance the growth of white poplar in unfavorable environmental conditions. To this end, for the first time, we performed whole-genome sequencing of 14 bacterial strains isolated from the tissues of the roots of white poplar in different geographical locations. We then performed a bioinformatics search to identify genes that may be useful for poplar growth and resistance to environmental pollutants and pathogens. Almost all endophytic bacteria obtained from white poplar roots are new strains of known species belonging to the genera *Bacillus*, *Corynebacterium*, *Kocuria*, *Micrococcus*, *Peribacillus*, *Pseudomonas*, and *Staphylococcus*. The genomes of the strains contain genes involved in the enhanced metabolism of nitrogen, phosphorus, and metals, the synthesis of valuable secondary metabolites, and the detoxification of heavy metals and organic pollutants. All the strains are able to grow on media without nitrogen sources, which indicates their ability to fix atmospheric nitrogen. It is concluded that the strains belonging to the genus *Pseudomonas* and bacteria of the species *Kocuria rosea* have the best poplar growth-stimulating and bioaugmentation potential, and the roots of white poplar are a valuable source for isolation of endophytic bacteria for possible application in ecobiotechnology.

## 1. Introduction

White poplar (*Populus alba* L.) is a fast-growing, medium-sized deciduous tree with a wide geographical range. It is considered an attractive plant for the green economy, as it provides a cheap source of raw materials and wood products, including cellulose [1] and sugars [2], which in turn can be used for biofuel production [1]. In addition, white poplar has a high potential for phytoremediation for areas subjected to strong anthropological impact [3,4], so it is often used for urban greening.

The problems of increasing plant resistance to adverse conditions of large settlements and industrial centers and increasing their productivity remain topical. The use of agrochemicals for these purposes may be limited both by legislation and from the position of possible impact on the environment, including soil and nearby water resources. Bioaugmentation of poplar trees using microorganisms can be used as an alternative and safe way of solving these problems [5,6]. This strategy, in turn, requires the study of the poplar microbiome and the characterization of its individual species, which, at present, are part of an intensively developing field in the study of plant–microbe interactions.

Most scientific research focuses on microorganisms living on the surface of poplar roots, including the surrounding layer of soil, called the rhizosphere [7,8]. In addition to surface-inhabiting microorganisms, there are endophytic microorganisms, such as bacteria, that inhabit the internal tissues of poplars and do not cause disease or other negative effects on the plant [9]. Each of the 300,000 plant species that exist on Earth is thought to be a host for endophytic bacteria [10]. However, only a small fraction of plants has endophytic bacteria characterized.

Bacterial endophytes colonize the same ecological niches in the plant as phytopathogens, making them a promising agent for biocontrol of phytopathogens [11]. Despite their benefits to plants, in some cases, they can act as pathogens themselves [12,13,14]. Endophytes are able to synthesize a wide range of metabolites that help it to win the competition for an ecological niche, while at the same time increasing the plant’s resistance [15,16]. In this context, the study of bacteria is useful to discover new potential antiviral [17] and antibacterial compounds [18] that could be used to enhance plant and human well-being [19]. In addition to plant growth-promoting properties, endophytes can be utilized in the process of bioaugmentation, i.e., detoxification or degradation of chemical environmental pollutants [20,21,22], thereby contributing to the phytoremediation of cities and industrial areas.

For most poplars, endophytes are not well studied, and to date, many gaps remain, both in terms of their diversity, ecology, and molecular genetic determinants. Fungal and bacterial endophytes have been described for species such as *P. trichocarpa* [14,23], *P. deltoides* [24], *P. euphratica* [25], *P. tremula* [26], *P. tomentosa*, *P. nigra*, and *P. canadensis* [27], as well as from some economically valuable hybrids [28,29,30,31].

For *P. alba*, widely distributed in Eurasia as a common member of the genus, we found no studies describing in detail the species specificity of root endophytes. However, the objects for search and study of poplar endophytes are mainly artificially bred hybrids with pronounced economically valuable potential, rather than pure white poplar lines [28,32]. However, the physiology, ecology, and habitus of poplar hybrids may differ significantly from plants growing in natural populations, which may significantly affect the distribution and species diversity of endophytes, as well as their potential benefits to the plant.

In this study, we obtained 14 pure strains of bacterial endophytes from the roots of wild poplar trees of different sexes and geographically distant regions. The cultivated strains for this study were selected based on their ability to grow rapidly on simple nutrient medium. For the first time, we sequenced and annotated the genomes of bacterial endophytes of white poplar. For a preliminary comprehensive assessment of the potential for plant growth stimulation and bioaugmentation of endophytes, their genomes were characterized for the presence of genes associated with the detoxification of heavy metals and organic compounds, as well as genes associated with the assimilation of nitrogen, phosphorus, and other nutrient sources that promote plant growth. Strains were also tested for their ability to grow on media without nitrogen sources. In addition, we assessed the biosafety of the strains by characterizing genes relevant to pathogenesis and resistance to viruses and antibiotics. As a result of comparative analysis of endophyte genomes, the most promising strains for enhancing poplar growth and development and its adaptation to adverse growing conditions were identified.

## 2. Materials and Methods

### 2.1. The Isolation of Culturable Bacterial Endophytes from P. alba Roots

**Figure 1:**
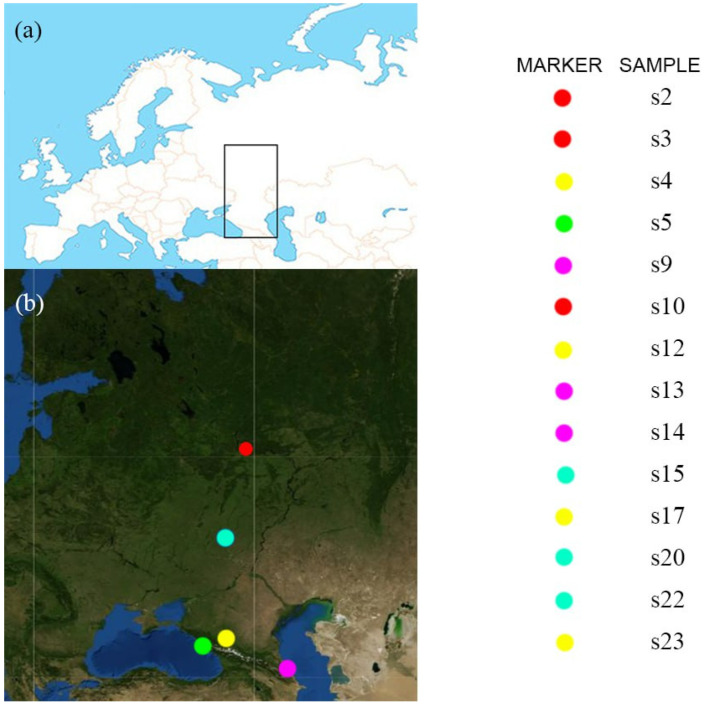
(**a**). Area of European Russia and the Russian Caucasus where root and rhizosphere samples were collected. (**b**). Labeled populations for collection of root samples.

Roots of adult trees of white poplar were collected in May 2023 in different geographical locations of the European part of Russia (Figure 1, Table 1). The obtained plant roots were placed in zip-lock bags and transported to the laboratory at +4 °C for further analysis within 24 h after collection.

**Figure 2:**
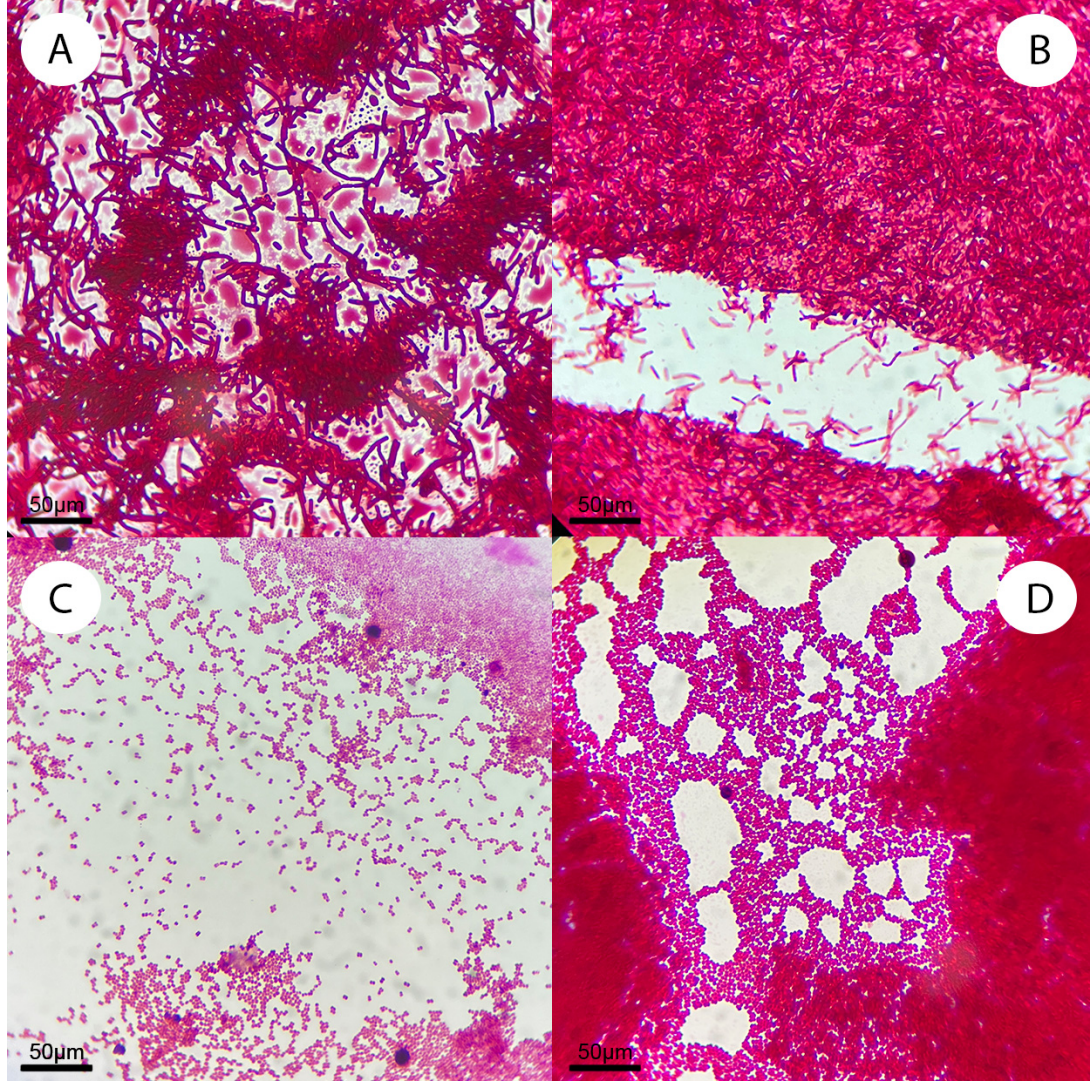
Microscopy of white poplar bacterial endophytes. Photos for several strains are given: (**A**)—s4, (**B**)—s23, (**C**)—s13, (**D**)—s22. Fuchsin staining. Staining magnification 100×.

For isolation of culturable bacteria, the roots were cut into small pieces and sterilized using surfactants and disinfectants: Tween-20 and sodium hypochlorite 3%, after application of which the root was thoroughly washed with autoclaved water. To confirm complete sterilization of the roots’ surface after disinfection, the roots were incubated in a Petri dish with sterile agarized Luria–Bertani (LB) nutrient medium for three days. In cases of colony development on such dishes, the roots were discarded. The roots were then ground in 1 mL of phosphate–salt buffer. The resulting suspension was serially diluted and aseptically poured 0.1 mL into sterile Petri dishes with fresh LB agar medium, then incubated at 20 °C for 48–72 h, and colony growth was checked. Microphotographs of several isolated strains are shown in Figure 2. Strains were preserved in 30% (*v*/*v*) glycerol at −80 °C until further analysis. 

Cultured bacterial endophyte strains of white poplar were selected based on several morphological differences of colonies (color, size, colony morphology, and color change of LB nutrient medium); we selected pure cultures of fast-growing and undemanding bacteria for further molecular studies.

### 2.2. Sex Determination of Plants from Which Bacterial Endophytes Were Obtained

Phenotypic differences (presence of sex-specific generative organs) were used to determine the sex of white poplar individuals and confirmed by PCR for the presence of the sex-specific gene *ARR17* in the sex-determining region using primers for *P. alba* developed for phylogenetic analysis [33].

### 2.3. Testing the Ability of Bacterial Strains to Grow on Nutrient Media without Nitrogen Sources

The ability of bacteria to fix free nitrogen was tested by surface seeding of colonies on Ashby’s agarized nutrient medium with addition mannitol or sucrose, Ashby’s agarized nutrient medium with addition sucrose, and Dobereiner’s agarized nutrient medium with and without addition malic acid. Visual assessment of colony growth was carried out on day 7. All experiments were carried out at the same time in three technical repetitions.

### 2.4. DNA Isolation, Library Preparation, and Whole-Genome Sequencing

Before DNA isolation, bacterial samples were stored at −80 °C. Total DNA was isolated from bacteria using the DNeasy PowerSoil Pro DNeasy kit (Qiagen, Hilden, Germany) according to the manufacturer’s protocol. DNA was quantified using a Qubit^®^2.0 fluorometer (Thermo Fisher Scientific, Waltham, MA, USA); quality control was performed on a NanoDrop^®^ ND-1000 spectrophotometer (NanoDrop Technologies Inc., Wilmington, DE, USA). The A260/A280 ratio in the DNA samples was 1.8–2.0. Approximately 100 ng of genomic DNA was fragmented into 500-bp-long double-stranded fragments using the Covaris S220 system (Covaris Inc., Woburn, MA, USA). Double-stranded DNA library was prepared using the NEBNext Ultra II DNA Library Prep Kit for Illumina (New England Biolabs, Ipswich, MA, USA) according to the manufacturer’s recommendations. AMPure XP beads were used to select DNA libraries by size (500–600 bp). Sample quantitation at standard concentration dilution to 4 nM was determined using a Qubit^®^2.0 fluorometer (Thermo Fisher Scientific, Waltham, MA, USA) and verified on an Agilent 2100 Bioanalyzer using a High Sensitivity DNA Kit (Agilent Technologies, Santa Clara, CA, USA). Genomic DNA of bacteriophage PhiX was used as an internal control. Sequencing was performed using the MiSeq Reagent Kit v3 (600 cycles) on Illumina’s MiSeq platform according to the manufacturer’s instructions. This resulted in 400 Mb of data (2 × 300 bp paired-end reads) for each sample. 

### 2.5. Endophyte Genome Assemblies and Annotation

First, Illumina reads were trimmed from the trailing 3′-end with both fixed quality threshold (30) and sliding window (4:17), filtered for average read quality (20), and adaptors were removed using the trimmomatic 0.39 tool [34]. PhiX reads were then removed by mapping to the bacteriophage reference genome (GCF_000819615.1) and its recircularized version (another breakpoint) using the bowtie2 2.3.5.1 tool [35] with increased sensitivity. Genomes were assembled using the SPAdes 3.15.4 assembler [36] with the specified k-mer sizes (21, 33, 47, 47, 55, 77, 99, 127); other parameters were set by default. Since the quality of the reads dropped significantly toward the 3′-end, the reads were cropped to the length at which average quality was preserved. The presence of erroneous k-mers in the dataset could lead to an excessive increase in assembly size, so we made sure that the sizes of the resulting assemblies did not exceed the corresponding references from NCBI Genome database.

Assembly statistics were calculated using QUAST 5.2.0 program [37]. The following parameters were evaluated: total assembly length, largest contig length, N50, auN50, L50, %GC. The assembled contigs were also screened with kraken 2.1.2 tool [38] (MaxiKraken database version 1903 was used) for contaminants (including PhiX, human), which were removed, as they conflict with PGAP annotation.

The resulting assemblies were annotated: (a) using the DFAST web service [39] and (b) using the standalone version of the NCBI Prokaryotic Genome Annotation Pipeline (PGAP; 2023-05-17.build6771; [40]). Using DFAST, the closest strain (type or non-type) was identified and the average nucleotide identity (ANI) was calculated. 

Assembly completeness and contamination with third-party prokaryotes was evaluated based on the presence and redundancy of single copy orthologous genes using both BUSCO 5.4.7 [41] and CheckM (embedded in the DFAST annotation pipeline) [42] tools. For BUSCO, we used various datasets (bacteria_odb10 as well as corresponding lower-level datasets: actinobacteria_phylum odb10, alphaproteobacteria odb10, bacillales odb10, bacilli odb10, corynebacteriales odb10, firmicutes odb10, gammaproteobacteria odb10, micrococcales odb10, proteobacteria odb10, pseudomonadales odb10, sphingomonadales odb10). The assembly completeness was considered as the maximum value among all datasets except the common dataset bacteria_odb10. The contamination (percentage of duplicated BUSCOs) was assessed in a similar manner with the difference that the bacteria_odb10 dataset was also included.

Genes responsible for nitrogen, phosphorus, metal metabolism, heavy metal tolerance and organic pollutant biodegradation were searched manually based on the annotation results. Identification of secondary metabolite biosynthesis gene clusters was performed using the antiSMASH (antibiotics & Secondary Metabolite Analysis Shell) bacterial version web service [43]. Antiphage systems were searched using the PADLOC (The Prokaryotic Antiviral Defense LOCator) web server [44]. Prediction of antibiotic resistance and pathogenic phenotypes was performed using the BV-BRC (Bacterial and Viral Bioinformatics Resource Center) Integrated Genome Analysis pipeline [45] integrated into the BV-BRC server. This analysis includes the use of the CARD (The Comprehensive Antibiotic Resistance Database) RGI (Resistance Gene Identifier) [46], PATRIC (Pathosystems Resource Integration Center) [47], NDARO (National Database of Antibiotic Resistant Organisms) [48], DrugBank [49], TTD (Therapeutic Target Database) [50], TCDB (The Transporter Classification Database) [51], VFDB (Virulence Factor Database) [52], and Victors (a knowledge base of virulence factors in human and animal pathogens) [53] databases.

### 2.6. Determining Taxonomic Affiliation

Taxonomic classification of the sequenced bacterial strains was determined based on the genome assemblies in two ways: First, using the Genome Taxonomy Database (GTDB) classifier [54] with own phylogenetically consistent taxonomy, which differs from the common NCBI taxonomy. Analysis was performed both via DFAST web service and GTDB-tk 2.3.2, which uses more recent GTDB release 217. Second, taxonomic classification was determined using the Type Strain Genome Server (TYGS) [55], which searches against a curated database of type strains with verified origins (corresponding to common taxonomy). In addition, a simple manual NCBI BLAST search (default database limited to Bacteria) was performed for several randomly selected genomic regions of 2–10 Kb and for several marker genes (*cyoA/B/C/D*).

## 3. Results

### 3.1. Overall Characteristics of Sequenced Genomes of Endophytic Bacteria

For 14 strains, the assembly sizes varied between 2.5 and 6.9 Mb and were in good agreement with the sizes of reference genomes of the corresponding bacterial species in the NCBI Genome database (Table 2). 

For 14 strains, the average N50 value was 253 Kb (range 33–570 Kb; average L50 = 10), BUSCO completeness in almost all assemblies > 99%, and the number of duplicated BUSCOs 4% or less (average 1.5%) (Table 3). Although the genome assemblies we obtained are not chromosome-level and are only contig-level, this should be sufficient to identify the vast majority of secondary metabolite biosynthesis gene clusters because the typical size of such clusters is 15–60 Kb [38], which is much smaller than the N50 value for most assemblies. Genome annotation was performed using the PGAP pipeline and DFAST web service, which implements the Prokka annotation tool [39]. NCBI Assembly accession numbers are provided in Supplementary Table S3.

The results of taxonomic classification of the assembled endophyte genomes obtained using the GTDB classifier agree with the results obtained using the TYGS service for the majority of strains (Table 3, Figures S1–S14, Tables S1 and S2). Some discrepancies in species identification were observed in the case of five strains. Strains s15 and s20 appear to belong to the genus *Pseudomonas*, but species assignment is complicated (in both GTDB and TYGS) due to low similarity to reference genomes. Strain s14 was classified as B. *bombysepticus* by GTDB and *B. cereus* by TYGS. The discrepancy was due to the fact that *B. cereus* strain FORC087, which has the highest similarity (ANI = 98.52%) to s14, was reclassified as *B. bombysepticus* in GTDB due to the highest similarity with the genomes of other *B. bombysepticus* strains. Strain s4 was classified by GTDB as *P. castrilensis*, but TYGS classified it as *P. frigoritolerans*. This discrepancy can be explained by the high similarity between the genomes of *P. castrilensis* and *P. frigoritolerans*. A similar situation was observed with strain s9, which is identified as *M. luteus* using both classifiers, but has higher ANI values with the type strains *M. aloeverae* and *M. yunnanensis*, which are not separated from *M. luteus* species in the latest GTDB 217 release. In general, the endophyte strains obtained during the present study belong to three phyla: Bacillota, Actinomycetota and Pseudomonadota (Table 4). The identified species are commonly found in the rhizosphere microbiomes of different poplar species.

Most of the cultivated endophytes were obtained from male plants (Table 5). At the same time, a few bacterial species were obtained from trees of both sexes, namely *B. cereus* and *P. frigoritolerans*. These data indirectly, due to the small volume of cultivated strains obtained, suggest a possible sex-specificity of endophyte distribution, since most bacteria were obtained from males. In general, sex-dependent differences in the rhizosphere community have been established previously [56,57]. Since the ANI results are <99.5% similarity to the closest related strains, we believe that the bacteria examined are new strains of known species. 

**Figure 3:**
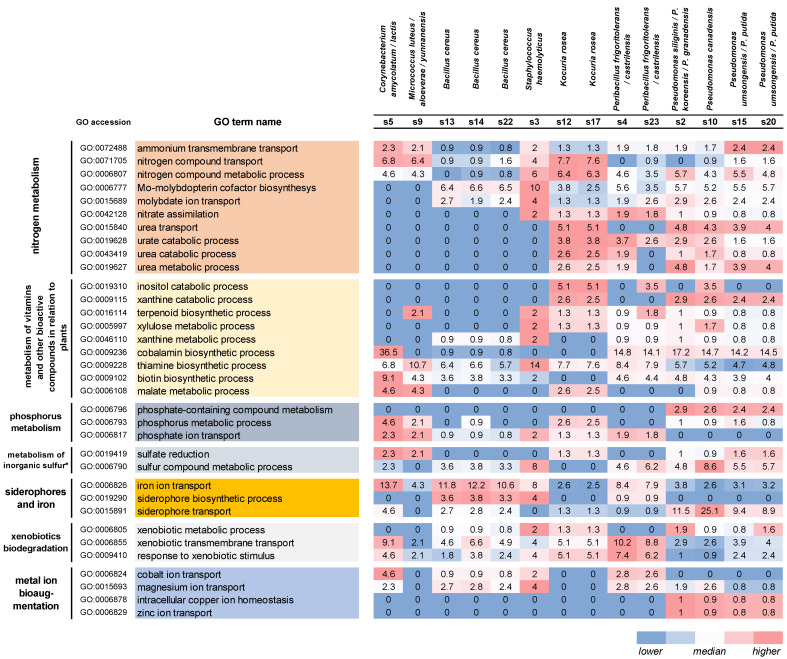
GO terms of white poplar endophyte genes associated with metabolic processes relevant to plant growth stimulation and biomonitoring. The table shows the number of genes (CDS) related to a current GO term and found in the assembled genomes. Since the total number of genes (CDS) varied significantly between strains (from 2.2 to 6.2 thousand), the reported number of genes was normalized to 5 thousand. The color scale reflects the relative number of genes in a given category (white—median value across all samples; blue—below the median; red—above the median). Comments: * Metabolism of inorganic sulfur from the point of view of potential further conversion into organics containing it.

To compare strains in the context of plant growth promotion and bioaugmentation capabilities, we performed a screening analysis for the presence of genes related to GO terms that are responsible for the metabolism of nitrogen, phosphorus, sulfur, iron, vitamins, and other bioactive compounds, as well as to the biodegradation of xenobiotics and bioaugmentation of metal ions (GO terms were fetched from the annotation). According to the results obtained (Figure 3), strains belonging to the genera *Peribacillus* and *Pseudomonas* possess genes belonging to almost all selected GO terms important for plant growth promotion. The lowest number of genes included in the considered GO terms was found in the genomes of members of the genera *Bacillus*, *Corynebacterium*, and *Micrococcus*. In the next section, this analysis was carried out in more detailed manner.

### 3.2. Genes and Biochemical Pathways That Promote Plant Growth and Soil Bioaugmentation

**Figure 4:**
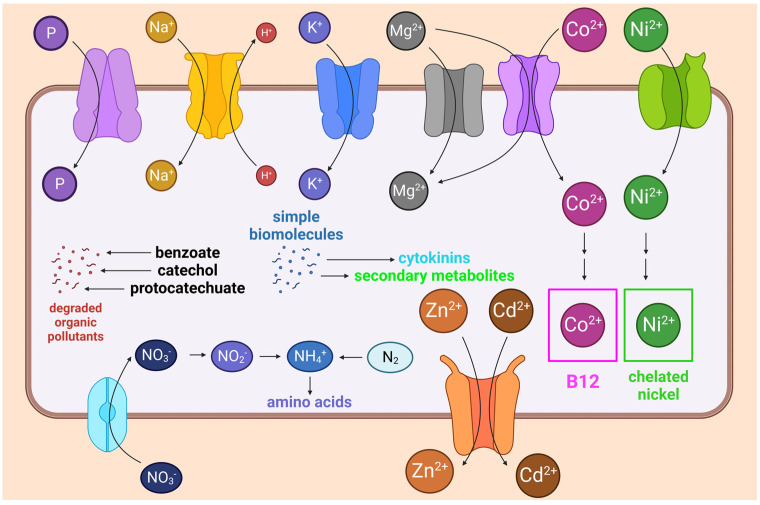
A simplified representation of the processes we have considered related to the metabolism of nitrogen, phosphorus, and metals (Co^2+^, Ni^2+^, Zn^2+^, and Cd^2+^), as well as the degradation of organic pollutants and the biosynthesis of bioactive compounds.

To evaluate the growth-stimulating potential of our strains, we searched for groups of genes related to nitrogen (transporters, nitrate and nitrite reduction, nitrogen fixation, Gln and Glu metabolism), phosphorus (phosphate transport, phosphonate decomposition), and metal (Na^+^, K^+^, Mg^2+^) assimilation, similar to what was carried out by Bartholomew et al. [58]. To assess the bioaugmentation potential of our strains, we examined genes encoding the influx, efflux, and chelation systems of heavy metal ions: Co^2+^, Ni^2+^, Zn^2+^, and Cd^2+^, using gene lists from the studies [6,59,60,61,62], etc.; and genes encoding enzymes for the biosynthesis of bioactive substances and biodegradation of organic pollutants: benzoates, catechols, and protocatechuate, according to the gene lists given in [63]. The results of the search are schematically summarized in Figure 4.

Most strains are capable of nitrate reduction. Nitrate transporter *narK* is present in all strains of *P. frigoritolerans.*, *Bacillus* spp., and *K. rosea*. *P. frigoritolerans* also have the assimilatory nitrate reductase *nasC*, whereas *Bacillus* spp., *K. rosea*, and *S. haemolyticus* have nitrate reductase subunits alpha, beta, and gamma, molybdenum cofactor assembly chaperone *narJ*, and hence are capable of nitrate respiration, and the latter also has nreAC regulators of nitrate respiration in its genome. In turn, strains of *Pseudomonas* spp. have nitrate reductase *napAE* (s15 and s20) and several other nitrate reductase types [64]. Thus, only strains s5 and s9 are probably incapable of nitrate reduction.

Most strains are able to reduce nitrite into ammonium. In the genomes of *Bacillus* spp. (s13, s22, s14), *S. haemolyticus* (s3), and *Pseudomonas* spp. (s2, s10, s15, s20), we found genes encoding both nitrite reductase large (*nirB*) and small (*nirD*) subunits, which means that they are able to reduce nitrite into ammonium. *Pseudomonas* spp. strains and *C. amycolatum* also possess a nitrite/sulfite reductase gene. *B. cereus* (s13, s14, and s22) and *P. frigoritolerans.* (s4 and s23) strains also possess a gene encoding ferredoxin-nitrite reductase. s4 and s23 strains also have *nasD* (encoding the NADPH-nitrite reductase) and *nirD* genes, whereas *K. rosea* strains (s12 and s17) have only the *nirB* gene. Strains s2, s3, s4, s10, s12, s15, s17, s20, and s23 also have a gene for the nitrate/nitrite transporter, while strains s2, s3, s5, s10, s12, s13, s14, s15, s17, s20, and s22 possess one to three genes encoding formate/nitrite transporter family protein. These data suggest that s12, s17, and especially s9 are probably able to assimilate nitrogen exclusively in the form of ammonium and amino acids.

Strains of *K. rosea* (s12 and s17), *Bacillus* spp. (s13, s22, s14), and *S. haemolyticus* (s3) also have *narH*, *narI*, *narJ* encoding nitrate reductase subunit genes, and *narK* encoding a nitrate transporter. Strain s3 also encodes the nitrate respiration regulation regulator (*nreC*) and sensor (*nreA*).

Next, genes for metabolism of the amino acids glutamine and glutamate were searched for, since they are the starting point for the assimilation of nitrogen into organic molecules in many organisms. The *glnAEHKKLPQRT* operon, which provides glutamine metabolism, was detected in all strains. The gene encoding glutamate ammonia ligase (*glnA*) is present in all strains, indicating its ability to metabolize ammonium. At the same time, other genes are present only in a part of strains. The *gltABCDPRSX* operon involved in glutamate metabolism was also detected in all strains. 

We also searched for genes encoding proteins involved in nitrogen fixation. In *Pseudomonas* spp. (s2, s10, s15, and s20), *P. frigoritolerans* (s4 and s23), and *M. luteus* (s9), only the gene encoding the gene for the hexameric Nif3-like dinuclear metal center protein is present in the genome, and there are no known genes for classical (canonical or alternative) nitrogenases. These data suggest that either this Nif3-like protein or as-yet-unknown proteins are involved in nitrogen fixation. *B. cereus* strains (s13, s22, and s14) have three copies of the gene encoding the Nif3-like protein as well as a gene for the NifU family Fe-S cluster assembly protein. Previously, a similar set of genes was found in the genome of *B. cereus* T4S, which is capable of nitrogen fixation [58]. *C. amycolatum* (s5) and *K. rosea* (s12 and s17) possess genes encoding Nif3-like protein and the SUF-system of NifU family proteins, and *S. haemolyticus* s3 encodes the SUF-system, NifU protein, and NifU N-terminal domain-containing protein, which confirms the ability of these strains to fix atmospheric nitrogen with the involvement of classical nitrogenases.

Next, we searched for genes involved in phosphorus metabolism. All strains possess the *pstSCAB* operon with varying degrees of integrity, which encodes an ABC-transporter that absorbs phosphates. Genes encoding PstA and PstB proteins were found in all strains. All strains except for s2, s15, and s20 contain genes encoding for PstC, and all actinomycetes (*Micrococcus, Kocuria, and Corynebacterium*) and *Pseudomonas* strains have a gene for PstS. Many of them also had *pho*-regulatory components: genes encoding the phosphate signaling complex protein PhoU were found in all strains studied: *phoR* in *Pseudomonas* spp (s2, s10, s15, and s20), *Bacillus cereus* (s13, s22, s14), and *P. frigoritolerans* (s4 and s23); *phoB* in s2, s10, s15, s20, s4, and s23, *phoP* in s13, s22, s14, s4, and s23; *phoD* and *phoE* only in s4 and s23.

Genes for the *phnABCEFNPWX* operon, which is involved in the degradation of phosphonates [65] and thus in phosphorus acquisition by other ways, have been found in many strains. The *pnhA* gene was found in all *Bacillaceae* strains (s4, s23, s13, s14, s22); *phnB* and *phnN* in all *Pseudomonas* (s2, s10, s15, s20); *phnC* in s3, s4, s13, s14, s22; *phnE* in s3, s4, s13, s14, s22, s2, s15, and s20; *phnF* only in s4 and s23; *pnhW* in all *Pseudomonas* and *Bacillaceae* strains; and *pnhX* only in s4 and s14. All strains studied except s3 also encode a phosphate acetyltransferase.

We further searched for ion channels and transporters that may mediate heavy metal tolerance. The vast majority of strains (s2, s3, s4, s10, s13, s14, s15, s20, s22) have one or more genes encoding the Na^+^/H^+^ antiporters NhaA, NhaB, or NhaC. All *Pseudomonas* strains encode the transcriptional activator NhaR, which regulates sodium import. Strain s3 stands out from other strains because, in addition to the genes described above, it has two copies of the *mnhBCDEFG* operon encoding the Na^+^/H^+^-antiporters Mnh1 and Mnh2.

Most strains (*Pseudomonas* spp. s2, s10, s15, s20; *Bacillus* spp. s13, s22, s14; and *C. amycolatum* sp. s5) contain genes encoding KdpA, KdpB, and KdpC subunits of the potassium-transporting ATPase. Strains from the genus *Bacillus* (s22, s13, s14) additionally encode a KdpD-like non-kinase potassium sensor. Strains of *P. frigoritolerans*. (s4 and s23) encode the KbfO subunit of the potassium channel.

All sequenced endophytic strains contain genes encoding the Mg^2+^/Co^2+^ transporter CorA. Most strains, with the exception of s9, s12, and s17, also have two or three genes encoding the magnesium transporter MgtE. *Pseudomonas* spp. (s2, s10, s15, s20) also encode the Co^2+^/Mg^2+^ efflux protein ApaG; s2 and s10 as well as *B. cereus* (s13, s22, s14) encode the Mg^2+^-translocating ATPase P-type MgtA. The presence of Co^2+^/Mg^2+^ transporters indicates the ability of strains to pump cobalt inside the cell, and possibly to biosynthesize B12 and other Co^2+^-containing compounds. Indeed, only strains of *Pseudomonas* spp. (s2, s10, s15, s20), *P. frigoritolerans* (s4, s23), and *C. amycolatum* s5 contain genes responsible for cobalamin biosynthesis. *Pseudomonas* spp. have the cobADFGJMNOTUW operon and three to four other genes encoding enzymes for vitamin B12 biosynthesis. *P. frigoritolerans* strains have the operon cobADIKMOSTU from the same biochemical pathway for B12 biosynthesis and the operon cbiBCQ from another pathway for B12 biosynthesis. Strain s5 has operons cobACFGMNOT and cbiQ, while the other strains have only the cobA gene for the enzyme responsible for the biosynthesis of precorrin-2, the precursor of siroheme [59], or none at all, as in the case of *K. rosea* strains s12 and s17.

As mentioned above, poplars are considered promising plants in terms of phytoremediation of soils from heavy metals, including nickel. In this regard, it is interesting to evaluate the role of poplar endophytic bacteria in this process. Strains s4 and s23 belonging to the genus *P. frigotolerans* encode nickel ABC-transporter proteins (*nikA* and *nikB* genes [60]). Moreover, these strains also encode LarB and LarE proteins involved in the biosynthesis of the nickel-coordinating cofactor for lactate racemase [61]. The genomes of *K. rosea* strains s12 and s17 encode nickel/cobalt transporter genes of the HoxN/HupN/NixA family, and strain s2 encodes an ABC nickel transporter.

Zinc and cadmium are closely related transition metals that are frequent soil contaminants. Strain s3 probably has a high tolerance to cadmium and zinc because its genome encodes CadD, CzcD, and CzrB transporters that carry out Cd^2+^, Zn^2+^/Cd^2+^, and Zn^2+^ efflux, respectively [6,62]. Strains s9, s12, and s17 also encode cadmium resistance transporters. All strains of *K. rosea* and *Bacillus* spp. also encode a heavy metal resistance metalloenzyme of the ArsI/CadI family. Although *Pseudomonas* spp. strains do not contain cadmium resistance genes, they have great potential in soil remediation from zinc: each of the strains s2, s10, s15, and s20 contains the *znuABC* operon responsible for the transport of Zn^2+^ ions inside the cell, as well as genes for the zinc transporter ZntB and the Cd(II)/Pb(II)-responsive transcriptional regulator CadR, which modulates the response to heavy metal exposure [6].

### 3.3. Ability of Poplar Endophytes to Grow on Nutrient Medium without a Nitrogen Source

To confirm the results of the bioinformatics search on the potential ability of endophytes to fix free nitrogen from the air, we tested by growing strains on solid nutrient medium containing no additional nitrogen sources. If the strains were able to fix nitrogen, they are able to form colonies on nitrogen-free nutrient medium. Surprisingly, our results (Table 6) indicate that all strains are able to grow on nitrogen-free media. Almost all colonies grew on Ashby’s medium (only s14 grew in the presence of mannitol). In contrast, more than half of the strains tested (s2, s3, s10, s12, s14, s15, s20, s22, s23) grew on Dobereiner’s medium without the addition of malic acid. Malic acid, when added to Dobereiner’s nutrient medium, inhibited nitrogen fixation in all bacterial strains studied. It is likely that the presence of mannitol in Ashby’s medium increases the efficiency of atmospheric nitrogen uptake or ensures its incorporation into biochemical reactions. At the same time, sodium molybdate, based on the same logic of reasoning, in Dobereiner medium does not contribute to the efficiency of nitrogen fixation, since bacteria predominantly grow on Ashby medium with a similar mineral composition. Given this observation, we are inclined to speculate that it is the carbon source or the presence of other organic compounds around the bacteria that they incorporate into their metabolic pathways that have a significant effect on the efficiency of nitrogen fixation. 

### 3.4. Secondary Metabolite Biosynthesis Clusters

We used AntiSMASH to search for genes encoding enzymes for the biosynthesis of secondary metabolites that may be useful for poplar, such as metal chelators or precursors of plant-important metabolites [43]. We considered more than a 60% match with the database to be satisfactory, since we took into account the possible variability of coding sequences and the structure of gene clusters. The search results are summarized in Table 7.

*K. rosea* appears to be able to metabolize ε-Poly-L-lysine. This compound is a homopolyamino acid characterized by the presence of a peptide bond between the carboxyl and ε-amino groups of L-lysine and has strong antimicrobial activity by interacting with phospholipids and consequently destabilizing the membrane [66]. Although this compound has antifungal activity, higher concentrations are required for this effect to occur [67]. Application of this substance is indeed able to limit the growth of potential pathogens of both fungal [67] and bacterial nature [68].

*B. cereus* was found to have genes for the biosynthesis of another potential antibacterial compound, thermoactinoamide A. It had an inhibitory effect on the growth of *S. aureus* culture [69]. Also, according to the results of the analysis, *P. frigoritolerans* synthesizes a poorly studied molecule, coranimine, which is a cyclic heptapeptide having nematocidal activity [70].

One of the most useful biosynthetic pathways for plants we have found in bacteria is the siderophore synthesis pathway. Siderophores are structurally diverse natural substances chelating metals, in particular Fe(II)/Fe(III), and possessing high affinity with them [71]. Free and bioavailable Fe(II) is rapidly oxidized to Fe(III), which is much less soluble, thus losing its bioavailability to plants despite its abundance in soils [72]. Of the 14 genomes we analyzed, siderophore biosynthesis pathways were found in 7. At the same time, the diversity of the presented compounds is very small: these are staphyloferrin A (*S. haemolyticus*), bacillibactin (*B. cereus*, *B. bombysepticus*), petrobactin (*B. cereus* and *B. bombysepticus*), schizokinen (*P. frigoritolerans*). Alluvial soils, on which white poplars most often grow under natural conditions, are characterized by low content of available forms of iron due to wide development of the processes of ogleying [73]. In this regard, colonization of their roots with bacteria capable of synthesizing siderophores is justified to increase iron bioavailability. 

*M. luteus* has the ability to biosynthesize carotenoids according to the AntiSMASH search results, but it was not possible to determine which ones. Carotenoids are a broad class of compounds synthesized by both microorganisms and plants, and play important roles in both plant life and human consumption [74]. Carotenoids play an important role in photosynthesis by implementing a photoprotective effect called non-photochemical quenching to safely utilize sunlight [75], in addition, these compounds stabilize [76]. 

A second interesting compound with potential biological activity is the lassopeptide paeninodin, which is putatively synthesized by *P. frigoritolerans* strains s4, s 23. Lassopeptides are synthesized ribosomally and posttranslationally modified, and have a filamentous topology similar to the lariat node. They undergo numerous modifications, such as phosphorylation, methylation, acetylation, etc., which is probably related to their functions [77]. The metabolic pathways involved in the synthesis of this compound were previously detected by the genomic analysis of plant endophytes [78], while antibacterial activity was previously shown for lassopeptides [79], for example, for paeninodin A [80]. In general, the biological functions of this intriguing class of compounds are poorly understood today.

Based on obtained data, we believe that in the case of planting white poplar on soils poor in iron in a digestible form, the use of strains belonging to the phylum Bacillota (*B. cereus*, *P. frigoritolerans*, *S. haemolyticus*) may be effective. Of note, strains s12 and s17 (*K. rosea*) can presumably act as a biocontrol element to prevent the development of phytopathologies.

### 3.5. Antiviral Defense Systems of Poplar Endophytic Bacteria

Bacteria are constantly exposed to phages, which act as an important element of selection and lead to the emergence and proliferation of various defense systems in bacterial genomes [81,82]. The study of these systems has previously led to the discovery of restriction endonucleases, which have found applications in molecular cloning and restriction analysis [83], as well as the CRISPR/Cas systems used for genome editing [84]. Interest in bacterial antiviral systems is growing and leading to the discovery of new systems [85,86]. To assess the presence and diversity of antiphage defense systems in our strains, we used the PADLOC service, which contains a large database of defense systems of various bacteria [44]. The antiviral defense systems we found are summarized in Table 8.

Some of the endophytic strains have restriction-modification (RM) systems. In such systems, the restriction component is needed to destroy foreign (e.g., phage) DNA, and the modification component is needed to protect bacteria’s own DNA [87]. *P. frigoritolerans* s4 and *M. luteus* s9 possess RM type I; RM type II is present in the *S. haemolyticus* s3, *M. luteus* s9 (as IIG type), and *C. amycolatum* s5; while RM type IV was identified only in the *B. cereus* s13 and s22, *P. frigoritolerans* s23, and in the s9 and s5 genomes. Both *K. rosea* strains (s12 and s17) and all *Pseudomonas* strains (s2, s10, s15, s20) lacked restriction-modification systems.

CRISPR/Cas systems are analogous to adaptive immunity in bacteria. The interest in CRISPR/Cas systems is due to the fact that most modern genome editors are based on their class II representatives, especially on Cas9 and Cas12 proteins. The disadvantages of existing editors encourage the search for new ones [88]. Only strain *P. frigoritolerans* s4, has a complete CRISPR/Cas type I-B1 system. Thus, our strains are unlikely to be a source of potentially novel genome editors.

Among other defensive systems, one of the most widely spread is the PD-T4-6 one. The PD-T4-6 protein, which is the only known component of this system, is present in the genomes of *B. cereus* strains (s13, s22, s14, all in 2 copies), *P. frigoritolerans* (both s4 and s23), *K. rosea* (both s12 and s17), *C. amycolatum* s5, and *Pseudomonas* spp. (s10 and s2). *P. siliginis* s2 has, in addition to PD-T4-6, a related system, PD-T4-7. PD-T4-6, PD-T4-7, and several other systems were recently discovered in the *E. coli* pangenome; they confer resistance against P2-like prophages and their mechanism of action is not currently understood [89]. However, our study hints at the possibility of widespread dissemination of these types of systems in the bacterial world.

The defense-associated reverse transcriptase (DRT) system appeared to be relatively common, especially among Pseudomonadota phylum representatives. DRT class I was detected in the genomes of *S. haemolyticus* s3 and *Pseudomonas* spp. (s2 and s20), DRT class II was found in the s2 strain, and DRT type III was found in *P. canadensis* s10 [90]. 

The dXTPase system exploits a vulnerability of phages in that they require a large number of nucleotides to replicate. There are two variations of this system: deoxycytidine triphosphate (dCTP) deaminase, which converts dCTP into dUTP, and deoxyguanosine triphosphatase (dGTPase), which converts dGTP into deoxyguanosine; in both cases, the cell becomes unsuitable for further phage multiplication [91]. We found that the dGTPase system is present only in Actinomycetes s9, s12, s17, and s9 strains.

The Cyclic-oligonucleotide-Based Anti-phage Signaling System (CBASS) is also common among *Actinomycetes*: we found it in s9, s12, and s17. For s9, it is the CBASS type I system. The CBASS is suicidal and works to prevent phages from spreading in a population by killing an individual cell. It consists of a cyclase that loops nucleotides to form signaling di- and trinucleotides and an effector that senses them and causes cell death, most often by embedding itself in the membrane to forming a pore [92]. 

Some components of the Defense Island System Associated with Restriction-Modification were detected in endophytic strains. *Pseudomonas* spp. strains s2, s15, and s20 possess the *drmC* gene encoding a phospholipase C subunit, while strains s15 and s17 possess the *drmA* and *drmB* genes encoding helicase and another phospholipase C subunit, respectively [93]. 

Although we have not found a complete BREX system in any of the strains, we were able to detect the gene *brxHI*, which is a gene encoding a putative helicase that is associated with a type 2 BREX, or Plg system [94], in the genomes of *Pseudomonas* spp. s2, s15, and s20 strains.

The Wadjet type III system was detected in *B. cereus* strains s13 and s22, while type I was found in *M. luteus* s9 strain. Wadjet is a four-gene system *jetABCD*, with *jetA*, *jetB,* and *jetC* being homologous to the housekeeping system of *mukBEF* encoding condensins, which ensures chromosome segregation, and *jetD* is homologous to topoisomerase VI gene. The entire Wadjet system is antiplasmid rather than antiphage [85]. 

The argonaute system, which is also known in eukaryotes, was restricted to *B. cereus* (s13, s22, and s14). Argonaute proteins are a very common antiviral defense system, originally appearing in prokaryotes and passed on to plants and humans. Most Arg proteins contain a PIWI domain, and they can in an RNA- or DNA-guided manner degrade RNA or DNA, causing abortive infection and often cell death [95]. 

The SoFic system appeared to be quite common among the strains studied: we found it in several strains of the Actinomycetota (s9, s5) and Pseudomonadota (s2, s15, s20) clades. The system consisting of a single SoFic protein is currently poorly understood, but this protein is known to contain a Fic domain capable of ligating AMP onto proteins [86].

The Mokosh Type II system was present in three Bacilli (s13, s22, s14) strains. The Mokosh Type II system consists of a single protein MkoC, which contains an RNA-helicase domain at the N-terminus and a nuclease domain at the C-terminus and probably performs its function by destroying phage RNA or RNA-DNA complexes [86]. 

AbiD was found in the genomes of s15, s20, and s9, while s9 also possesses a system AbiE. They both cause abortive infection. AbiD consists of a single protein, AbiD1, whose mRNA translation is induced by the phage protein Orf1 [96], while AbiE is a type IV toxin–antitoxin system and consists of the antitoxin AbiEi and the toxin AbiEii [97].

In addition, we found the following defensive systems: septu type I (in s2, s10, and s4), Lamassu (in s22 and s14), Tiamat (in s14), PT DndFGH (in s4), SspABCD and PT SspFGH (in s23), RosmerTA (in s9), mza and ppl (in s12 and s17), HEC-06 (in s23 and s5), GAO19 (in s10), Uzume (in s4 and s9), kiwa (in s10), tmn (in s10), SEFIR (in s2), Azaza and Dpd (in s15), gabija (in s20). Lamassu and SEFIR are both suicidal systems: Lamassu consists of the proteins LmuA, LmuB, and LmuC, with LmuB serving as a sensor and LmuA as an effector causing cell death via DNA degradation and NAD+ depletion [86]. RosmerTA is a toxin–antitoxin (TA) system with a Zn-peptidase antitoxin RsmA and a toxin RsmT [86]. Although gabija, kiwa, septu, Uzume, and Tiamat were described, their mechanism of action is currently poorly understood [85,86].

Very short patch repair (VSPR) is not a defense system but often works in conjunction with restriction-modification systems and provides repair of O6-methylguanine (O6mG):T mismatches. VSPR includes the methyltransferase proteins, MutH, MutS, MutL, or Vsr [98]. We found it in strains s4 and s2, with s2 lacking RM systems but having a number of other defensive systems.

### 3.6. Pathogenic Potential and Antibiotic Resistance of Poplar Endophytic Bacteria

The last important aspect of the bioaugmentation potential of bacterial strains is the safety assessment in terms of human pathogenicity and the spread of antibiotic resistance (AMR) genes. Genes potentially involved in virulence and AMR were searched using a number of resources, including CARD RGI [46], PATRIC [47], NDARO, DrugBank, TTD, TCDB, PATRIC_VF, VFDB, Victors, and the complex genome analysis pipelines on the BV-BCR server [45]. The number of targets found by each program is shown in Table 9.

#### 3.6.1. Pathogenicity of Poplar Endophytes to Humans

Endophytic strains of *B. cereus* (s13 and s22) and *B. bombysepticus* (possibly, s14) belong to the *B. cereus* group of closely related species. *B. cereus* can lead to food poisoning and is also able to cause wounds, mucosal infections, and systemic diseases [99]. *B. bombysepticus* is known as an entomopathogen and has been proposed for use as a biopesticide [99]; its pathogenicity to humans has not been reported. All these strains have a full set of genes of a three-component pore-forming complex called nonhemolytic enterotoxin (NHE), which is encoded by genes nheA, nheB, and nheC as well as all three genes hblA, hblC, and hblD, encoding subunits B, L2, and L1 of the hemolysin BL (HBL) pore complex. The genomes of all three strains also contain genes encoding cytotoxin K (*cytK*), also known as hemolysin IV; immune inhibitor A (*inhA*), required to avoid the host immune response; and three superoxide dismutases (*sodA1, sodA2*, and *sodC*) that help cells survive oxidative stress, including the host immune response [100]. They also possess the genes encoding anthrolysin O, which is a thiol-activated cytolysin. Thus, strains s13, s22, and s14 have some pathogenic potential. 

Endophytic strains of Actinomycetes, *P. frigoritolerans*, *S. haemolyticus*, *M. luteus*, *K. rosea*, and *C. amycolatum* do not have genes that could be directly involved in human pathogenic processes.

Strains s2, s10, s15, and s20 belong to the genus *Pseudomonas*, which includes free-living representatives as well as plant (such as *P. fluorescens* and *P. syringae* strains) and human (such as *P. aeruginosa*) pathogens. The genomes of s2, s10, s15, and s20 strains contain genes with only indirect relevance to pathogenesis, such as genes *algA*, *algI*, and *alg8* encoding alginate biosynthesis enzymes; genes *algB* and *algR* encoding regulators of their expression; and *algU* encoding the RNA polymerase RpoE sigma factor, which is also involved in alginate biosynthesis regulation. Strain s10 also possesses the type VI secretion system TssB-TssC. However, we did not detect either the exotoxin A gene or components of the pathogenic type 3 secretion system. Our data indicate that endophytic *Pseudomonas* strains have low, if any, pathogenic potential.

#### 3.6.2. AMR Genes

One group of antibiotic resistance genes is antibiotic inactivation enzymes, which we found in the all genomes of all six strains of Bacilli class. The genes encoding the chloramphenicol O-acetyltransferase (*catA*) superfamily and fosfomycin resistance protein (*fosB*) are present in all strains of *Bacillus* spp. (s13, s14, and s22) and *P. frigoritolerans* (s4 and s23), while the gene encoding beta-lactamase of the BcII family is present only in *Bacillus* spp. and *S. haemolyticus* s3 possesses *blaZ*, *lnuA*, and *mphC* family genes, encoding beta-lactamase, lincosamide nucleotidyltransferase, and macrolide 2′-phosphotransferase enzymes, respectively.

We also examined the presence of genes encoding antibiotic target protection proteins. *Bacillus* spp. (s13, s22, s14) possess genes encoding undecaprenyl-diphosphatase (*bcrC*) and ABC-F type ribosomal protection protein (*lsaB*), while *S. haemolyticus* s3 has genes encoding ABC-F type ribosomal protection protein (*msrA*).

Another important category is genes encoding ABC transporters that confer multidrug resistance to bacteria. Among the studied strains belonging to the actinomycetes class, they were found only in *M. luteus* (s9). Strain s9 has the *macB* gene encoding a macrolide-specific ABC transporter. All *Bacilli* strains (s13, s22, s14, s4, s23, and s3) contain genes encoding the YkkCD transporter. In addition, *Bacillus* spp. strains encode the BcrAB system, and *P. frigoritolerans* and *S. haemolyticus* encode the BceAB system. We also found *mdtABC-TolC* (in s13), *lmrB* (in s4), and *norA* (in s3) genes. *Pseudomonas* spp. have the highest number of genes encoding ABC transporters (mrAB-TolC, MacAB-OprM/TolC, MdtABC-TolC, TriABC-TolC, MexAB-OprM, MexEF-OprN, MexJK-OprM/TolC pumps, and the MexVW-OprM system). Moreover, *Pseudomonas* spp. strains have the largest number of porin genes including *oprF*, *oprB*, *oprD* family, the *occD* subfamily (*occD1*, *occD2*, *occD3*, *occD6* and *occD4* (in s10) or *occD7* (in s2, s15 and s20)) and *occK* subfamily (*occK8* in all strains, *occK1* and *occK10* in s15 and s20, *occK5* in s10 and *occK9* in s20).

## 4. Discussion

### 4.1. Ecology of the Studied Bacteria: Representation in Soils, Rhizosphere, and Endophytes of Other Plants

Most root bacterial endophytes originate from the rhizosphere community [32], and a small fraction of root endophytes transfer to the phyllosphere (e.g., leaves, stems) through the plant vascular system [101]. The rhizosphere community is numerous, diverse, and dynamic, and is highly dependent on climatic conditions and soil type [7,102], as previously identified for *P. trichocarpa* [103]. 

All of the bacterial species we identified in this study are common representatives of soil microorganisms and have previously been found as plant endophytes, but little or no data are available for members of the poplar genus. At the same time, we observed that the majority of the obtained cultivated strains belonged to males of white poplar, despite the fact that the roots of plants of both sexes were similarly selected beforehand. In this study, with a high degree of probability, all the bacteria studied turned out to be new natural strains of known species. This observation allows us to consider white poplar roots as a source of new natural bacterial strains with potential ecobiotechnological value for biostimulation of woody plant growth. 

*B. cereus* was multiple times reported to be an endophytic species. Comprehensive analysis of the genome of strain T4S isolated from sunflower plant revealed that it can promote plant uptake of nitrogen, phosphorus and iron as well as biosynthesize auxins and cytokinins [58]. A strain isolated from mustard (*Sinapis*) secretes chitinase and is able to protect plant roots from putrefactive fungi [104]. Other strains of this species produce chitosanase, alkaline serine, and neutral proteases, and thus have a repellent and toxic effect on the phytopathogenic nematode *Meloidogyne incognita* [105]. And a strain isolated from *Garcinia xanthochymus* has even been used to synthesize antibacterial silver nanoparticles [106].

The members of *Peribacillus* genus has been previously identified as an endophyte of pine, and genetically close strains Q2H1 and *P. castrilensis* as growth-promoting bacteria in potato and tomato, respectively [107,108]. One of the most interesting features of *Peribacillus* sp. is the production of coranimine, which exhibits nematocidal activity against *Bursaphelenchus xylophilus*, the causative agent of pine wilt disease [109]; clusters of coranimine biosynthesis are also present in our strains.

*S. haemolyticus* is not only a component of the human skin microbiome and its opportunistic pathogen, but has also been isolated from willow stems of *Salix viminalis × S. miyabeana* hybrids [110] and described as an endophyte of rice seeds and tomato roots; the ability to colonize plant tissues and adapt to this environment appears to be through horizontal gene transfer, and pathogenicity factors may be lost through this specialization [111].

*K. rosea* (s12 and s17) has been previously described as an endophyte of *Rehmannia glutinosa* [112], ryegrass (*Lolium perenne*) [113], and annual wormwood (*Atremisia annua*) [114].

*C. amycolatum*, like *M. luteus*, is a widespread bacterium and one of the dominant organisms on the human skin [115]. However, it has been described as an endophytic microorganism associated with banana shoot tips [116].

Representatives of the genus *Pseudomonas*, to which 4 of our 14 strains belong, have been reported many times as plant endophytes. It is important to note that 21 endophytic species of *Pseudomonas* sp. were previously examined in the study of the root microbiome of *P. deltoides*, but they were not identified to species [117]. *P. canadensis* was originally identified in the field as a biocontrol agent, showing antagonism towards phytopathogenic fungi [118].

We believe that the detection of these bacterial species in other plant taxa as endophytes is an appropriate validation of our data that may indirectly rule out contamination of Petri dishes by foreign bacteria during the preparation of biological samples.

### 4.2. Metabolism of Nitrogen, Phosphorus, and Metals and Tolerance to Heavy Metals

White poplar endophytes are well equipped with genes encoding various transporters for obtaining mineral and organic sources of nitrogen and phosphorus from the soil. Because phosphorus is essential for poplar physiology, these data suggest that poplar may obtain phosphorus in part through bacterial metabolism. The ability of endophytes to fix atmospheric nitrogen is also very useful for poplar, as bacteria can provide it with available forms of nitrogen and thus stimulate its growth even in nutrient-poor soils. The observed potential ability to incorporate metals into metabolism makes the identified bacteria interesting from the position of a factor contributing to soil bioaugmentation, including as a means of enhancing the accumulation of heavy metals directly by the plant. Since white poplar is considered as a plant capable of phytoremediation, it is important to consider bacteria that, being in a positive relationship with poplar, can induce synergism and enhance the accumulation of heavy metals through their transport into plant tissues.

Based on the obtained data, we believe that representatives of the genus Pseudomonas (s2, s10, s15, s20) and K. rosea strains (s12 and s17) are the most promising for improving the metabolism of mineral compounds and metals.

### 4.3. Biodegradation of Organic Pollutants

In addition to purifying soils from polluting metals such as cobalt, nickel, zinc, and cadmium, poplars can be utilized in the process of soil purification from organic pollutants. We performed a brief search for catechol (*cat*), benzoate (*ben*), and protocatechuate (*pca*) degradation genes, similar to a previous study [63]. The genomes of *Pseudomonas* spp. strains showed the greatest potential for bioaugmentation: while strain s2 has only the *pcaCDFGHR* operon encoding proteins for protocatechol degradation, strain s10 also has the *catAC* operon for catechol degradation, and strains s15 and s20, in addition to the *pcaCDFGHQR* and *catAC* operons, carry the *benABC* operon for benzoic acid degradation. *K. rosea* strains s12 and s17, carrying the *benABC*, *catABC*, and *pcaCGH* operons, are also promising bioaugmentation agents. As for the other strains, we found *catAR* and *pcaF* genes in strain s4, but only *catAR* in s23; *pcaD* in s5; *pcaBGH* in s9; *catA* only in *Bacillus* spp. s13, s14, and s22; and nothing in s3.

Given that endophytes are most often bacteria that are initially part of the rhizosphere community, the potential for biodegradation of organic compounds may contribute to the bioaugmentation of soils from anthropogenic pollutants, which is relevant for plants growing in highly urbanized areas; at the same time, the degradation products of pollutants may serve as a carbon source for the rhizosphere bacterial consortium. Undoubtedly, the ability to decompose organic pollutants should be confirmed by further studies, but the preliminary results are intriguing.

Anyway, most of the strains are capable of biodegradation of pollutants. However, we consider strains belonging to the genus Pseudomonas (s2, s10, s15, s20) and Kocuria (s12, s17) to be the most promising for degradation of organic pollutants, since they have a larger number of genes that could potentially be involved in this process.

### 4.4. Ability to Biosynthesize Secondary Metabolites

A number of endophytic bacteria have the potential to synthesize biologically active molecules that limit colonization of poplar by other organisms. This is another strategy of bacteria to survive in plant tissues and allow them to win in competition. In order for bacteria not to be perceived by the plant as pathogens, they need to develop a strategy in which benefits are possible for both the endophytic bacterium and the host plant. As a rule, bacteria produce compounds with antibacterial and antifungal activity [119], and the list of these connections is very wide. The ability to biosynthesize secondary metabolites likely enhances success in competing for an ecological niche. This explains the broad representation of biosynthesis pathways for siderophores and other compounds with activity directed against other living organisms.

The ability to synthesize biologically active compounds is primarily beneficial to the host plant: these compounds promote both plant growth, as in the case of siderophores, and its defense against root pathogens. This is an example of mutually beneficial cooperation: the endophytic bacterium receives a convenient environment with low competition for nutrients, and poplar, in turn, receives the opportunity to use the metabolites synthesized by the bacterium in their own life process.

### 4.5. Antiviral Defense Systems

Our data show that each endophytic strain possesses several defense systems. This means that endophytic strains are not only well protected against phages, but may also be resistant to horizontal gene transfer. In terms of adaptation to environmental conditions, this can be a disadvantage. However, we are talking about endophytic strains living in plant tissues, where the strength of natural selection factors may be reduced, and, therefore, the influence of horizontal gene transfer on bacterial survival is of less importance.

### 4.6. Safety Issues of White Poplar Endophytic Bacteria

We have attempted to assess the risk of pathogenicity of endophytic strains to humans and the risk of AMR gene spread. This can be taken into account when considering these strains for bioaugmentation. We have not attempted to predict the phytopathogenic potential of these strains for other plants, as this is an extremely difficult task due to a lack of understanding of the mechanisms. However, it is known that plants, including poplars, utilize symbiotic relationships with potentially phytopathogenic microorganisms to suppress environmental competitors [14]. All our predictions are made exclusively via the bioinformatic approach and should be further validated experimentally. Our data suggest that only strains of the *B. cereus* group (s13, s14, and s22) may have pathogenic potential for humans. However, the presence of pathogenicity or AMR genes is not a guarantee that they are functionally active.

## 5. Conclusions

In this work, we first isolated and characterized 14 endophyte strains of wild-growing white poplar belonging to three different bacterial phyla. We assembled genomes of all 14 strains and revealed the presence of genes that may be responsible for the bioaugmentation properties of endophytes. Most of these genes may assist in the uptake of nitrogen, phosphorus, and biochemically useful metals by poplar, and enhance the synthesis of beneficial secondary metabolites that contribute to poplar resistance to heavy metals and organic pollutants. All the strains were able to grow on a nutrient medium without nitrogen, indicating their role in stimulating plant growth by nitrogen fixation. For several strains belonging to the genus *Pseudomonas* (s15 and s20), we hypothesized membership in a previously undescribed and potentially new species. Other strains obtained have not been previously described, which allows us to consider white poplar roots as a natural source of potentially useful plant growth-biostimulating bacteria. It is likely that most of the strains studied are safe for humans, but these data require further investigation. Based on the data presented, we consider bacteria of the genus *Pseudomonas* (strains s2, s10, s15, s20) and species of *K. rosea* (strains s12 and s17) to be the most interesting and promising for further study of their effect on poplar growth. These strains are presumably safe and are able to incorporate metals into poplar metabolic pathways and synthesize a number of bioactive compounds. We hope that the primary data we obtained on endophytic bacterial strains of white poplar may be of interest to researchers and may be further used in the development of biological agents to improve poplar growth, as well as for bioaugmentation of contaminated soils.

## Figures and Tables

**Table 1:** Coordinates of the location of trees from which samples were obtained.

Strain	Location
s2	56.349087, 44.059552
s3	56.352357, 44.059899
s4	44.027473, 43.06353
s5	43.491915, 39.896625
s9	41.8821, 48.534351
s10	56.346653, 44.061317
s12	43.954701, 42.765658
s13	41.881878, 48.534617
s14	41.880614, 48.528303
s15	50.793133, 41.979933
s17	43.954701, 42.765658
s20	50.786221, 41.986017
s22	50.789744, 41.988373
s23	44.027603, 43.067865

**Table 2:** Genome assembly statistics of endophytic bacteria.

Strain	Number of Contigs (>1 Kb)	Largest Contig, Kb	Total Length, Mb	GC (%)	N50, Kb	auN, Kb	L50	Reference Genome Length, Mb
s2	59	774.2	5.90	60.0	202.6	293.0	9	6.5
s3	47	388.7	2.47	32.8	98.3	140.0	7	2.6
s4	45	605.5	5.59	40.6	316.5	306.4	7	5.6
s5	38	342.7	2.50	58.9	153.0	158.7	6	2.5
s9	40	359.6	2.52	72.8	128.3	172.2	6	2.5
s10	38	768.5	6.29	60.3	450.6	420.9	6	6.4
s12	79	303.6	4.17	72.2	182.4	138.6	10	4
s13	29	877.4	5.41	35.3	570.2	541.5	4	5.8
s14	30	788.5	5.31	35.1	451.8	466.1	4	5.8
s15	98	395.1	6.88	60.3	159.3	163.0	15	6.2 *
s17	197	122.0	4.19	72.2	33.3	44.1	35	4
s20	144	269.2	6.72	60.4	99.7	100.6	25	6.2 *
s22	199	765.7	5.85	35.0	302.8	324.4	7	5.8
s23	89	941.0	5.67	40.2	185.3	298.0	8	5.6

* for some strains deposited to the NCBI Assembly, genome sizes were greater than 7 Mb.

**Table 3:** Statistics on annotation and completeness assessment of sequenced genomes of bacterial endophytes.

Strain	Number of CDSs	Number of rRNA Genes	Number of tRNA Genes	Completeness, CheckM (DFAST)	Completeness, BUSCO	Contamination, CheckM (DFAST)	Contamination, BUSCO	Taxonomic Classification (GTDB (G), TYGS (T))	Closest Type Strains (or Species Representatives), ANI
s2	5203	4	66	100.00%	100.00%	0.19%	0.27%	*Pseudomonas siliginis* (G *, T *)	*P. siliginis* SWRI31 [96.46%]
s3	2481	4	49	99.80%	99.67%	0.21%	0.00%	*Staphylococcus haemolyticus* (G, T)	*S. haemolyticus* NCTC 11042 [97.13%]
s4	5449	6	67	99.35%	100.00%	0.66%	0.81%	*Peribacillus frigoritolerans* (G, T)	*P. frigoritolerans* FJAT-2396 [97.10%], DSM 8801 [96.81%]; *P. castrilensis* N3 LMG 32505 [98.28%]
s5	2198	3	53	99.49%	98.29%	0.41%	0.00%	*Corynebacterium amycolatum* (G *, T *)	*C. amycolatum “A”* SK46 ^§^ [95.23%]; *C. amycolatum* ATCC 49368 [94.83%]
s9	2299	3	52	98.54%	98.88%	0.23%	0.19%	*Micrococcus luteus* (G, T)	*M. luteus* ATCC 4698 [97.22%]; *M. aloeverae* DSM 27472 [98.26%]; *M. yunnanensis* DSM 21948 [98.16%]
s10	5718	4	59	100.00%	100.00%	0.35%	0.55%	*Pseudomonas canadensis* (G, T)	*P. canadensis* 2–92 [97.70%]
s12	3808	3	55	99.54%	99.07%	4.87%	1.69%	*Kocuria rosea* (G, T)	*K. rosea* ATCC 186 [99.37%]
s13	5448	8	56	99.22%	100.00%	0.59%	4.03%	*Bacillus cereus* (G, T)	*B. cereus* ATCC 14579 [98.26%]
s14	5267	6	59	99.22%	99.34%	0.79%	4.10%	*Bacillus bombysepticus* (G), *B. cereus* (T)	*B. cereus* ATCC 14579 [97.10%]; *B. cereus* FORC087 (reclassified into *B. bombysepticus* in GTDB) [98.52%]
s15	6159	3	55	81.04%	99.74%	12.99%	1.64%	*Pseudomonas* sp. (G) **	*P. putida “G”* ASAD ^§§^ [93.13%]; *P. umsongensis* DSM 16611 [91.19%]
s17	3817	3	53	99.54%	99.44%	5.37%	1.69%	*Kocuria rosea* (G, T)	*K. rosea* ATCC 186 [99.38%]
s20	6026	4	66	80.99%	99.45%	11.66%	1.65%	*Pseudomonas* sp. (G) **	*P. putida “G”* ASAD ^§§^ [93.19%]; *P. umsongensis* DSM 16611 [91.10%]
s22	5629	5	70	99.22%	99.67%	2.54%	4.07%	*B. cereus* (G, T)	*B. cereus* ATCC 14579 [98.18%]
s23	5352	6	71	99.35%	100.00%	3.58%	3.23%	*P. frigoritolerans* (G, T)	*P. frigoritolerans* FJAT-2396 [98.75%]; DSM 8801 [97.18%]; *P. castrilensis* N3 (reclassified into *P. frigoritolerans* in GTDB) [97.27%]

* Asterisks indicate cases of uncertain identification at the species level. For TYGS (T *), these are cases where precise species identification was not achieved, but type strains with sufficient homology (dDDH score) were available. For GTDB (G *), asterisks indicate cases where taxonomic classification was achieved by only one of two methods (either ANI or tree topology analysis). ** Possible new species. ^§^ This strain is a species representative for *Corynebacterium amycolatum “A”* subgroup in the GTDB systematics. ^§§^ This strain is a species representative for *Pseudomonas putida “G”* subgroup in the GTDB systematics.

**Table 4:** Taxonomic identity of the studied strains based on the NCBI Taxonomy database.

Phylum	Class	Order	Family	Genus	Species	Strain
Bacillota	Bacilli	Bacillales	Bacillaceae	*Bacillus*	*B. cereus*	s13
						s22
						s14
				*Peribacillus*	*P. frigoritolerans*	s4
						s23
			Staphylococcaceae	*Staphylococcus*	*S. haemolyticus*	s3
Actinomycetota	Actinomycetes	Micrococcales	Micrococcaceae	*Micrococcus*	*M. luteus.*	s9
				*Kocuria*	*K. rosea*	s12
						s17
		Mycobacteriales	Corynebacteriaceae	*Corynebacterium*	*C. amycolatum*	s5
Pseudomonadota	Gammaproteobacteria	Pseudomonadales	Pseudomonadaceae	*Pseudomonas*	*P. canadensis*	s10
					*P. siliginis*	s2
					*Pseudomonas* sp.	s15
						s20

**Table 5:** Sex of plants from which root endophyte strains were obtained and identified.

Strain	Bacterial Species	Sex of the Host Tree
s2	*P. siliginis*	Male
s3	*S. haemolyticus*	Female
s4	*P. frigoritolerans*	Male
s5	*C. amycolatum*	Female
s9	*M. luteus*	Male
s10	*P. canadensis*	Male
s12	*K. rosea*	Male
s13	*B. cereus*	Male
s14	*B. cereus*	Male
s15	*Pseudomonas* sp.	Male
s17	*K. rosea*	Male
s20	*Pseudomonas* sp.	Male
s22	*B. cereus*	Female
s23	*P. frigoritolerans*	Female

**Table 6:** The ability of white poplar endophytes to grow on nitrogen-free medium.

Strain	Ashby’s Mannitol Medium	Ashby’s Medium	Dobereiner’s Medium Malic Acid +	Dobereiner’s Medium Malic Acid −
s2	**+**	**+**	**−**	**+**
s3	**+**	**+**	**−**	**+**
s4	**+**	**+**	**−**	**−**
s5	**+**	**+**	**−**	**−**
s9	**+**	**+**	**−**	**−**
s10	**+**	**+**	**+**	**+**
s12	**+**	**+**	**−**	**+**
s13	**+**	**+**	**−**	**−**
s14	**+**	**−**	**−**	**+**
s15	**+**	**−**	**−**	**+**
s17	**+**	**+**	**−**	**−**
s20	**+**	**+**	**−**	**+**
s22	**+**	**+**	**−**	**+**
s23	**+**	**+**	**−**	**+**

**Table 7:** Clusters of secondary metabolite biosynthesis genes found in the genomes of endophytic strains.

Strain	Bacterial Species	Region	From	To	Type	Most Similar Known Cluster	Similarity
s13	*B. cereus*	Region 1.1	124,986	138,693	NI-siderophore	petrobactin	100%
		Region 9.1	45,152	96,900	NRP-metallophore	bacillibactin	85%
s22	*B. cereus*	Region 6.3	202,313	254,061	NRP-metallophore	bacillibactin	85%
		Region 14.1	9815	23,522	NI-siderophore	petrobactin	100%
		Region 134.1	1	1304	NRPS	thermoactinoamide A	100%
s14	*B. cereus*	Region 7.1	41,536	93,284	NRP-metallophore	bacillibactin	85%
		Region 14.1	9914	23,621	NI-siderophore	petrobactin	100%
s4	*P. frigoritolerans*	Region 1.2	537,557	553,069	NI-siderophore	schizokinen	60%
		Region 11.1	60,018	128,334	NRPS	koranimine	87%
s23	*P. frigoritolerans*	Region 3.1	66,600	126,919	NRPS	koranimine	87%
		Region 6.1	51,405	66,917	NI-siderophore	schizokinen	60%
		Region 14.1	42,776	66,671	lassopeptide	paeninodin	100%
s3	*S. haemolyticus*	Region 8.1	70,705	85,702	NI-siderophore	staphyloferrin A	100%
s9	*M. luteus*	Region 22.1	9041	23,211	terpene	carotenoid	66%
s12	*K. rosea*	Region 11.1	89,770	123,837	NAPAA	branched-chain fatty acid	100%
s17	*K. rosea*	Region 52.1	5871	26,881	NAPAA	ε-poly-L-lysine	100%
		Region 54.1	649	26,385	NAPAA	branched-chain fatty acid	66%

**Table 8:** The antiviral systems we detected in the genomes of the endophyte strains studied.

Strain	Bacterial Species	Count	Systems
s13	*B. cereus*	7	DMS, Mokosh TypeII, PD-T4-6 (×2), RM type IV, argonaute solo, wadjet type III
s22	*B. cereus*	10	DMS, Lamassu, Mokosh Type II, PD-T4-6 (×2), RM type IV, argonaute solo (×2), wadjet type III
s14	*B. cereus/bombysepticus*	7	Lamassu, Mokosh Type II, PD-T4-6 (×2), Tiamat, argonaute solo, argonaute type III
s4	*P. frigoritolerans*	8	PD-T4-6, PT DndFGH, RM type I, Uzume (×2), VSPR, cas type I-B1, septu type I
s23	*P. frigoritoleran*	5	HEC-06, PD-T4-6, PT SspABCD, PT SspFGH, RM type IV
s3	*S. haemolyticus*	3	DRT class I, RM type II (×2)
s9	*M. luteus*	16	AbiD (×2), AbiE, DMS (×2), Mokosh Type II, RM type I (×2), RM type IIG, RM type IV, RosmerTA, SoFic, Uzume, cbass type I, dXTPase, wadjet type I
s12	*K. rosea*	7	DMS (×2), PD-T4-6, cbass, dXTPase, mza, ppl
s17	*K. rosea*	7	DMS (×2), PD-T4-6, cbass, dXTPase, mza, ppl
s5	*C. amycolatum*	9	DMS other (×2), HEC-06, PD-T4-6, RM type II (×2), RM type IV, SoFic, dXTPase
s10	*P. canadensis*	8	DMS, DRT type III, GAO 19, PD-T4-6, kiwa (×2), septu type I, tmn
s2	*P.* *siliginis*	11	DMS (×3), DRT class I, DRT class II, PD-T4-6, PD-T4-7, SEFIR, SoFic, VSPR, septu type I
s15	*Pseudomonas* sp.	7	AbiD (×2), Azaca, DMS, Dpd, SoFic (×2)
s20	*Pseudomonas* sp.	7	AbiD (×2), DMS (×2), DRT class I, SoFic, gabija

**Table 9:** Number of virulence and AMR factors detected in the genomes of white poplar bacterial endophytes.

		Antibiotic Resistance			Drug Target		Transporter	Virulence Factor		
Strain	Bacterial Species	CARD	PATRIC	NDARO	DrugBank	TTD	TCDB	PATRIC_VF	VFDB	Victors
s13	*B. cereus*	7	49	4	28		47		6	11
s22	*B. cereus*	6	54	4	28		52	1	12	12
s14	*B. cereus/bombysepticus*	6	51	4	27		47		9	12
s4	*P. frigoritolerans*	1	42		13	1	14	2	1	3
s23	*P. frigoritolerans*.	1	45		14	2	16	3	4	5
s3	*S. haemolyticus*	15	40	8	26	10	45		1	18
s9	*M. luteus*	1	28		6	1	8	3	2	1
s12	*K. rosea*	1	42		2		3	1		
s17	*K. rosea*	1	42		2		4	1		
s5	*C. amycolatum*	1	23		3	1	5	4	2	3
s10	*P. canadensis*	3	72		27	6	73	1	27	25
s2	*P. siliginis*	5	66		28	8	69		24	28
s15	*Pseudomonas* sp.	2	85		32	6	66		26	24
s20	*Pseudomonas* sp.	3	85	1	37	7	68		25	25

## Data Availability

Whole-prokaryotic-genome sequencing data are deposited in the NCBI SRA with BioProject identifier PRJNA1040149. The genome assembly accession numbers are provided in the Supplementary Table S3. The other datasets generated during and/or analyzed during the current study are available from the corresponding author on reasonable request.

## Supplementary Materials

The following supporting information can be downloaded at: https://www.mdpi.com/article/10.3390/biology12121519/s1, Figure S1: Phylogenetic tree inferred by TYGS for type strains with highest genomic similarity scores (MASH algorithm) to s2, s10, s15, s20 strains; Figure S2: Phylogenetic tree inferred by TYGS for type strains with highest 16S similarity scores to s2, s10, s15, s20 strains; Figure S3: Phylogenetic tree inferred by TYGS for type strains with highest genomic similarity scores (MASH algorithm) to s3 strain; Figure S4: Phylogenetic tree inferred by TYGS for type strains with highest 16S similarity scores to s3 strain; Figure S5: Phylogenetic tree inferred by TYGS for type strains with highest genomic similarity scores (MASH algorithm) to s4, s23 strains; Figure S6: Phylogenetic tree inferred by TYGS for type strains with highest 16S similarity scores to s4, s23 strains; Figure S7: Phylogenetic tree inferred by TYGS for type strains with highest genomic similarity scores (MASH algorithm) to s5 strain; Figure S8: Phylogenetic tree inferred by TYGS for type strains with highest 16S similarity scores to s5 strain; Figure S9: Phylogenetic tree inferred by TYGS for type strains with highest genomic similarity scores (MASH algorithm) to s9 strain; Figure S10: Phylogenetic tree inferred by TYGS for type strains with highest 16S similarity scores to s9 strain; Figure S11: Phylogenetic tree inferred by TYGS for type strains with highest genomic similarity scores (MASH algorithm) to s12 and s17 strains; Figure S12: Phylogenetic tree inferred by TYGS for type strains with highest 16S similarity scores to s12 and s17 strains; Figure S13: Phylogenetic tree inferred by TYGS for type strains with highest genomic similarity scores (MASH algorithm) to s13, s14 and s22 strains; Figure S14: Phylogenetic tree inferred by TYGS for type strains with highest 16S similarity scores to s13, s14 and s22 strains; Table S1: Results of pairwise comparisons of genomes of s4, s23 and closest type strains (TYGS); Table S2: Results of pairwise comparisons of genomes of s9 strain and closest type strains (TYGS); Table S3: NCBI Assembly accession numbers of the genomic sequences.

## References

[1] Zhu J., Chen L., Gleisner R., Zhu J.Y. (2019). Co-production of bioethanol and furfural from poplar wood via low temperature (≤90 °C) acid hydrotropic fractionation (AHF). Fuel.

[2] Jiménez-López L., Martín-Sampedro R., Eugenio M.E., Santos J.I., Sixto H., Cañellas I., Ibarra D. (2020). Co-production of soluble sugars and lignin from short rotation white poplar and black locust crops. Wood Sci. Technol..

[3] Di Lonardo S., Capuana M., Arnetoli M., Gabbrielli R., Gonnelli C. (2011). Exploring the metal phytoremediation potential of three *Populus alba* L. clones using an in vitro screening. Environ. Sci. Pollut. Res. Int..

[4] Kostic O., Gajic G., Jaric S., Vukov T., Matic M., Mitrovic M., Pavlovic P. (2021). An Assessment of the Phytoremediation Potential of Planted and Spontaneously Colonized Woody Plant Species on Chronosequence Fly Ash Disposal Sites in Serbia-Case Study. Plants.

[5] Ciadamidaro L., Madejón P., Madejón E. (2014). Soil chemical and biochemical properties under *Populus alba* growing: Three years study in trace element contaminated soils. Appl. Soil Ecol..

[6] Vannucchi F., Imperato V., Saran A., Staykov S., D’Haen J., Sebastiani L., Vangronsveld J., Thijs S. (2021). Inoculated Seed Endophytes Modify the Poplar Responses to Trace Elements in Polluted Soil. Agronomy.

[7] Wu N., Li Z., Meng S., Wu F. (2021). Soil properties and microbial community in the rhizosphere of *Populus alba var. pyramidalis along a chronosequence*. Microbiol. Res..

[8] Simmer R., Mathieu J., da Silva M.L.B., Lashmit P., Gopishetty S., Alvarez P.J.J., Schnoor J.L. (2020). Bioaugmenting the poplar rhizosphere to enhance treatment of 1,4-dioxane. Sci. Total Environ..

[9] van der Lelie D., Taghavi S., Monchy S., Schwender J., Miller L., Ferrieri R., Rogers A., Wu X., Zhu W., Weyens N. (2009). Poplar and its bacterial endophytes: Coexistence and harmony. Crit. Rev. Plant Sci..

[10] Strobel G., Daisy B., Castillo U., Harper J. (2004). Natural products from endophytic microorganisms. J. Nat. Prod..

[11] Berg G., Eberl L., Hartmann A. (2005). The rhizosphere as a reservoir for opportunistic human pathogenic bacteria. Environ. Microbiol..

[12] DeMers M. (2022). Alternaria alternata as endophyte and pathogen. Microbiology.

[13] Brader G., Compant S., Vescio K., Mitter B., Trognitz F., Ma L.J., Sessitsch A. (2017). Ecology and Genomic Insights into Plant-Pathogenic and Plant-Nonpathogenic Endophytes. Annu. Rev. Phytopathol..

[14] Newcombe G., Fraser S.J., Ridout M., Busby P.E. (2020). Leaf Endophytes of *Populus trichocarpa* Act as Pathogens of Neighboring Plant Species. Front. Microbiol..

[15] Hagaggi N.S.A., Mohamed A.A.A. (2020). Plant-bacterial endophyte secondary metabolite matching: A case study. Arch. Microbiol..

[16] Matsumoto H., Fan X., Wang Y., Kusstatscher P., Duan J., Wu S., Chen S., Qiao K., Wang Y., Ma B. (2021). Bacterial seed endophyte shapes disease resistance in rice. Nat. Plants.

[17] Lacerda I., Polonio J.C., Golias H.C. (2022). Endophytic Fungi as a Source of Antiviral Compounds—A Review. Chem. Biodivers..

[18] Martinez-Klimova E., Rodriguez-Pena K., Sanchez S. (2017). Endophytes as sources of antibiotics. Biochem. Pharmacol..

[19] Burragoni S.G., Jeon J. (2021). Applications of endophytic microbes in agriculture, biotechnology, medicine, and beyond. Microbiol. Res..

[20] Doty S.L. (2008). Enhancing phytoremediation through the use of transgenics and endophytes. New Phytol..

[21] Kumar A., Tripti, Voropaeva O., Maleva M., Panikovskaya K., Borisova G., Rajkumar M., Bruno L.B. (2021). Bioaugmentation with copper tolerant endophyte *Pseudomonas lurida* strain EOO26 for improved plant growth and copper phytoremediation by *Helianthus annuus*. Chemosphere.

[22] Ma Y., Zhang C., Oliveira R.S., Freitas H., Luo Y. (2016). Bioaugmentation with Endophytic Bacterium E6S Homologous to *Achromobacter piechaudii* Enhances Metal Rhizoaccumulation in Host *Sedum plumbizincicola*. Front. Plant Sci..

[23] Doty S.L., Sher A.W., Fleck N.D., Khorasani M., Bumgarner R.E., Khan Z., Ko A.W., Kim S.H., DeLuca T.H. (2016). Variable Nitrogen Fixation in Wild *Populus*. PLoS ONE.

[24] Utturkar S.M., Cude W.N., Robeson M.S., Yang Z.K., Klingeman D.M., Land M.L., Allman S.L., Lu T.Y., Brown S.D., Schadt C.W. (2016). Enrichment of Root Endophytic Bacteria from *Populus deltoides* and Single-Cell-Genomics Analysis. Appl. Environ. Microbiol..

[25] Cheng G., Cheng Y., Rahman E. (2023). Diversity analysis of *Populus euphratica* endophytic bacteria in Tarim River Basin, China. PeerJ.

[26] Albrectsen B.R., Siddique A.B., Decker V.H.G., Unterseher M., Robinson K.M. (2018). Both plant genotype and herbivory shape aspen endophyte communities. Oecologia.

[27] Wang X., Wu G., Han S., Yang J., He X., Li H. (2023). Differentiation and Identification of Endophytic Bacteria from *Populus* Based on Mass Fingerprints and Gene Sequences. Int. J. Mol. Sci..

[28] Hanak A.M., Nagler M., Weinmaier T., Sun X., Fragner L., Schwab C., Rattei T., Ulrich K., Ewald D., Engel M. (2014). Draft Genome Sequence of the Growth-Promoting Endophyte *Paenibacillus* sp. P22, Isolated from *Populus*. Genome Announc..

[29] Gkorezis P., Van Hamme J.D., Bottos E.M., Thijs S., Balseiro-Romero M., Monterroso C., Kidd P.S., Rineau F., Weyens N., Vangronsveld J. (2016). Draft Genome Sequence of *Pantoea ananatis* GB1, a Plant-Growth-Promoting *Hydrocarbonoclastic* Root Endophyte, Isolated at a Diesel Fuel Phytoremediation Site Planted with *Populus*. Genome Announc..

[30] Li Y., Bian D.R., Chang J.P., Guo L.M., Yang X.Q. (2020). *Sphingomonas populi* sp. nov., isolated from bark of *Populus* × euramericana. Int. J. Syst. Evol. Microbiol..

[31] Kaldorf M., Koch B., Rexer K.H., Kost G., Varma A. (2005). Patterns of interaction between *Populus* Esch5 and *Piriformospora indica*: A transition from mutualism to antagonism. Plant Biol..

[32] Beckers B., Op De Beeck M., Weyens N., Boerjan W., Vangronsveld J. (2017). Structural variability and niche differentiation in the rhizosphere and endosphere bacterial microbiome of field-grown poplar trees. Microbiome.

[33] Kim G., Leite Montalvao A.P., Kersten B., Fladung M., Müller N. (2021). The genetic basis of sex determination in *Populus* provides molecular markers across the genus and indicates convergent evolution. Silvae Genet..

[34] Bolger A.M., Lohse M., Usadel B. (2014). Trimmomatic: A flexible trimmer for Illumina sequence data. Bioinformatics.

[35] Langmead B., Salzberg S.L. (2012). Fast gapped-read alignment with Bowtie 2. Nat. Methods.

[36] Bankevich A., Nurk S., Antipov D., Gurevich A.A., Dvorkin M., Kulikov A.S., Lesin V.M., Nikolenko S.I., Pham S., Prjibelski A.D. (2012). SPAdes: A new genome assembly algorithm and its applications to single-cell sequencing. J. Comput. Biol..

[37] Gurevich A., Saveliev V., Vyahhi N., Tesler G. (2013). QUAST: Quality assessment tool for genome assemblies. Bioinformatics.

[38] Wood D.E., Salzberg S.L. (2014). Kraken: Ultrafast metagenomic sequence classification using exact alignments. Genome Biol..

[39] Tanizawa Y., Fujisawa T., Nakamura Y. (2018). DFAST: A flexible prokaryotic genome annotation pipeline for faster genome publication. Bioinformatics.

[40] Tatusova T., DiCuccio M., Badretdin A., Chetvernin V., Nawrocki E.P., Zaslavsky L., Lomsadze A., Pruitt K.D., Borodovsky M., Ostell J. (2016). NCBI prokaryotic genome annotation pipeline. Nucleic Acids Res..

[41] Simao F.A., Waterhouse R.M., Ioannidis P., Kriventseva E.V., Zdobnov E.M. (2015). BUSCO: Assessing genome assembly and annotation completeness with single-copy orthologs. Bioinformatics.

[42] Parks D.H., Imelfort M., Skennerton C.T., Hugenholtz P., Tyson G.W. (2015). CheckM: Assessing the quality of microbial genomes recovered from isolates, single cells, and metagenomes. Genome Res..

[43] Blin K., Shaw S., Augustijn H.E., Reitz Z.L., Biermann F., Alanjary M., Fetter A., Terlouw B.R., Metcalf W.W., Helfrich E.J.N. (2023). antiSMASH 7.0: New and improved predictions for detection, regulation, chemical structures and visualisation. Nucleic Acids Res..

[44] Payne L.J., Meaden S., Mestre M.R., Palmer C., Toro N., Fineran P.C., Jackson S.A. (2022). PADLOC: A web server for the identification of antiviral defence systems in microbial genomes. Nucleic Acids Res..

[45] Olson R.D., Assaf R., Brettin T., Conrad N., Cucinell C., Davis J.J., Dempsey D.M., Dickerman A., Dietrich E.M., Kenyon R.W. (2023). Introducing the Bacterial and Viral Bioinformatics Resource Center (BV-BRC): A resource combining PATRIC, IRD and ViPR. Nucleic Acids Res..

[46] Alcock B.P., Huynh W., Chalil R., Smith K.W., Raphenya A.R., Wlodarski M.A., Edalatmand A., Petkau A., Syed S.A., Tsang K.K. (2023). CARD 2023: Expanded curation, support for machine learning, and resistome prediction at the Comprehensive Antibiotic Resistance Database. Nucleic Acids Res..

[47] Wattam A.R., Davis J.J., Assaf R., Boisvert S., Brettin T., Bun C., Conrad N., Dietrich E.M., Disz T., Gabbard J.L. (2017). Improvements to PATRIC, the all-bacterial Bioinformatics Database and Analysis Resource Center. Nucleic Acids Res..

[48] Feldgarden M., Brover V., Gonzalez-Escalona N., Frye J.G., Haendiges J., Haft D.H., Hoffmann M., Pettengill J.B., Prasad A.B., Tillman G.E. (2021). AMRFinderPlus and the Reference Gene Catalog facilitate examination of the genomic links among antimicrobial resistance, stress response, and virulence. Sci. Rep..

[49] Wishart D.S., Feunang Y.D., Guo A.C., Lo E.J., Marcu A., Grant J.R., Sajed T., Johnson D., Li C., Sayeeda Z. (2018). DrugBank 5.0: A major update to the DrugBank database for 2018. Nucleic Acids Res..

[50] Zhu F., Han B., Kumar P., Liu X., Ma X., Wei X., Huang L., Guo Y., Han L., Zheng C. (2010). Update of TTD: Therapeutic Target Database. Nucleic Acids Res..

[51] Saier M.H., Reddy V.S., Moreno-Hagelsieb G., Hendargo K.J., Zhang Y., Iddamsetty V., Lam K.J.K., Tian N., Russum S., Wang J. (2021). The Transporter Classification Database (TCDB): 2021 update. Nucleic Acids Res..

[52] Liu B., Zheng D., Zhou S., Chen L., Yang J. (2022). VFDB 2022: A general classification scheme for bacterial virulence factors. Nucleic Acids Res..

[53] Sayers S., Li L., Ong E., Deng S., Fu G., Lin Y., Yang B., Zhang S., Fa Z., Zhao B. (2019). Victors: A web-based knowledge base of virulence factors in human and animal pathogens. Nucleic Acids Res..

[54] Parks D.H., Chuvochina M., Rinke C., Mussig A.J., Chaumeil P.A., Hugenholtz P. (2022). GTDB: An ongoing census of bacterial and archaeal diversity through a phylogenetically consistent, rank normalized and complete genome-based taxonomy. Nucleic Acids Res..

[55] Meier-Kolthoff J.P., Carbasse J.S., Peinado-Olarte R.L., Goker M. (2022). TYGS and LPSN: A database tandem for fast and reliable genome-based classification and nomenclature of prokaryotes. Nucleic Acids Res..

[56] Xia Z., He Y., Korpelainen H., Niinemets U., Li C. (2023). Allelochemicals and soil microorganisms jointly mediate sex-specific belowground interactions in dioecious *Populus cathayana*. New Phytol..

[57] Guo Q., Liu L., Liu J., Korpelainen H., Li C. (2022). Plant sex affects plant-microbiome assemblies of dioecious *Populus cathayana* trees under different soil nitrogen conditions. Microbiome.

[58] Adeleke B.S., Ayangbenro A.S., Babalola O.O. (2021). Genomic Analysis of Endophytic *Bacillus cereus* T4S and Its Plant Growth-Promoting Traits. Plants.

[59] Balabanova L., Averianova L., Marchenok M., Son O., Tekutyeva L. (2021). Microbial and Genetic Resources for Cobalamin (Vitamin B12) Biosynthesis: From Ecosystems to Industrial Biotechnology. Int. J. Mol. Sci..

[60] Eitinger T., Mandrand-Berthelot M.A. (2000). Nickel transport systems in microorganisms. Arch. Microbiol..

[61] Desguin B., Soumillion P., Hols P., Hausinger R.P. (2016). Nickel-pincer cofactor biosynthesis involves LarB-catalyzed pyridinium carboxylation and LarE-dependent sacrificial sulfur insertion. Proc. Natl. Acad. Sci. USA.

[62] Liu H., Zhang Y., Wang Y., Xie X., Shi Q. (2021). The Connection between Czc and Cad Systems Involved in Cadmium Resistance in Pseudomonas putida. Int. J. Mol. Sci..

[63] Rosas-Diaz J., Escobar-Zepeda A., Adaya L., Rojas-Vargas J., Cuervo-Amaya D.H., Sanchez-Reyes A., Pardo-Lopez L. (2021). *Paenarthrobacter* sp. GOM3 Is a Novel Marine Species With Monoaromatic Degradation Relevance. Front. Microbiol..

[64] Tan W., Liao T.H., Wang J., Ye Y., Wei Y.C., Zhou H.K., Xiao Y., Zhi X.Y., Shao Z.H., Lyu L.D. (2020). A recently evolved diflavin-containing monomeric nitrate reductase is responsible for highly efficient bacterial nitrate assimilation. J. Biol. Chem..

[65] Martin J.F., Liras P. (2021). Molecular Mechanisms of Phosphate Sensing, Transport and Signalling in Streptomyces and Related Actinobacteria. Int. J. Mol. Sci..

[66] Hyldgaard M., Mygind T., Vad B.S., Stenvang M., Otzen D.E., Meyer R.L. (2014). The antimicrobial mechanism of action of epsilon-poly-l-lysine. Appl. Environ. Microbiol..

[67] Shu C., Cui K., Li Q., Cao J., Jiang W. (2021). Epsilon-poly-l-lysine (epsilon-PL) exhibits multifaceted antifungal mechanisms of action that control postharvest Alternaria rot. Int. J. Food Microbiol..

[68] Rodrigues B., Morais T.P., Zaini P.A., Campos C.S., Almeida-Souza H.O., Dandekar A.M., Nascimento R., Goulart L.R. (2020). Antimicrobial activity of Epsilon-Poly-L-lysine against phytopathogenic bacteria. Sci. Rep..

[69] Teta R., Marteinsson V.T., Longeon A., Klonowski A.M., Groben R., Bourguet-Kondracki M.L., Costantino V., Mangoni A. (2017). Thermoactinoamide A, an Antibiotic Lipophilic Cyclopeptide from the Icelandic Thermophilic Bacterium *Thermoactinomyces vulgaris*. J. Nat. Prod..

[70] Ponpandian L.N., Rim S.O., Shanmugam G., Jeon J., Park Y.H., Lee S.K., Bae H. (2019). Phylogenetic characterization of bacterial endophytes from four Pinus species and their nematicidal activity against the pine wood nematode. Sci. Rep..

[71] Li K., Chen W.H., Bruner S.D. (2016). Microbial siderophore-based iron assimilation and therapeutic applications. Biometals.

[72] Miethke M., Marahiel M.A. (2007). Siderophore-based iron acquisition and pathogen control. Microbiol. Mol. Biol. Rev..

[73] Vodyanitskii Y.N., Shoba S.A. (2015). Biogeochemistry of carbon, iron, and heavy metals in wetlands (Analytical review). Mosc. Univ. Soil. Sci. Bull..

[74] Langi P., Kiokias S., Varzakas T., Proestos C. (2018). Carotenoids: From Plants to Food and Feed Industries. Methods Mol. Biol..

[75] Demmig-Adams B., Stewart J.J., Lopez-Pozo M., Polutchko S.K., Adams W.W. (2020). Zeaxanthin, a Molecule for Photoprotection in Many Different Environments. Molecules.

[76] Hashimoto H., Uragami C., Cogdell R.J. (2016). Carotenoids and Photosynthesis. Subcell. Biochem..

[77] Duan Y., Niu W., Pang L., Bian X., Zhang Y., Zhong G. (2022). Unusual Post-Translational Modifications in the Biosynthesis of Lasso Peptides. Int. J. Mol. Sci..

[78] Semenzato G., Alonso-Vasquez T., Del Duca S., Vassallo A., Riccardi C., Zaccaroni M., Mucci N., Padula A., Emiliani G., Palumbo Piccionello A. (2022). Genomic Analysis of Endophytic Bacillus-Related Strains Isolated from the Medicinal Plant *Origanum vulgare* L. Revealed the Presence of Metabolic Pathways Involved in the Biosynthesis of Bioactive Compounds. Microorganisms.

[79] Hegemann J.D., Zimmermann M., Xie X., Marahiel M.A. (2015). Lasso peptides: An intriguing class of bacterial natural products. Acc. Chem. Res..

[80] Lohans C.T., Huang Z., van Belkum M.J., Giroud M., Sit C.S., Steels E.M., Zheng J., Whittal R.M., McMullen L.M., Vederas J.C. (2012). Structural characterization of the highly cyclized lantibiotic paenicidin A via a partial desulfurization/reduction strategy. J. Am. Chem. Soc..

[81] Hampton H.G., Watson B.N.J., Fineran P.C. (2020). The arms race between bacteria and their phage foes. Nature.

[82] Safari F., Sharifi M., Farajnia S., Akbari B., Karimi Baba Ahmadi M., Negahdaripour M., Ghasemi Y. (2020). The interaction of phages and bacteria: The co-evolutionary arms race. Crit. Rev. Biotechnol..

[83] Di Felice F., Micheli G., Camilloni G. (2019). Restriction enzymes and their use in molecular biology: An overview. J. Biosci..

[84] Anzalone A.V., Koblan L.W., Liu D.R. (2020). Genome editing with CRISPR-Cas nucleases, base editors, transposases and prime editors. Nat. Biotechnol..

[85] Doron S., Melamed S., Ofir G., Leavitt A., Lopatina A., Keren M., Amitai G., Sorek R. (2018). Systematic discovery of antiphage defense systems in the microbial pangenome. Science.

[86] Millman A., Melamed S., Leavitt A., Doron S., Bernheim A., Hor J., Garb J., Bechon N., Brandis A., Lopatina A. (2022). An expanded arsenal of immune systems that protect bacteria from phages. Cell Host Microbe.

[87] Phillips Z.N., Husna A.U., Jennings M.P., Seib K.L., Atack J.M. (2019). Phasevarions of bacterial pathogens—Phase-variable epigenetic regulators evolving from restriction-modification systems. Microbiology.

[88] Shmakov S., Smargon A., Scott D., Cox D., Pyzocha N., Yan W., Abudayyeh O.O., Gootenberg J.S., Makarova K.S., Wolf Y.I. (2017). Diversity and evolution of class 2 CRISPR-Cas systems. Nat. Rev. Microbiol..

[89] Patel P.H., Maxwell K.L. (2023). Prophages provide a rich source of antiphage defense systems. Curr. Opin. Microbiol..

[90] Gao L., Altae-Tran H., Bohning F., Makarova K.S., Segel M., Schmid-Burgk J.L., Koob J., Wolf Y.I., Koonin E.V., Zhang F. (2020). Diverse enzymatic activities mediate antiviral immunity in prokaryotes. Science.

[91] Tal N., Millman A., Stokar-Avihail A., Fedorenko T., Leavitt A., Melamed S., Yirmiya E., Avraham C., Brandis A., Mehlman T. (2022). Bacteria deplete deoxynucleotides to defend against bacteriophage infection. Nat. Microbiol..

[92] Millman A., Melamed S., Amitai G., Sorek R. (2020). Diversity and classification of cyclic-oligonucleotide-based anti-phage signalling systems. Nat. Microbiol..

[93] Ofir G., Melamed S., Sberro H., Mukamel Z., Silverman S., Yaakov G., Doron S., Sorek R. (2018). DISARM is a widespread bacterial defence system with broad anti-phage activities. Nat. Microbiol..

[94] Goldfarb T., Sberro H., Weinstock E., Cohen O., Doron S., Charpak-Amikam Y., Afik S., Ofir G., Sorek R. (2015). BREX is a novel phage resistance system widespread in microbial genomes. EMBO J..

[95] Koopal B., Potocnik A., Mutte S.K., Aparicio-Maldonado C., Lindhoud S., Vervoort J.J.M., Brouns S.J.J., Swarts D.C. (2022). Short prokaryotic Argonaute systems trigger cell death upon detection of invading DNA. Cell.

[96] Bidnenko E., Chopin A., Ehrlich S.D., Chopin M.C. (2009). Activation of mRNA translation by phage protein and low temperature: The case of Lactococcus lactis abortive infection system AbiD1. BMC Mol. Biol..

[97] Dy R.L., Przybilski R., Semeijn K., Salmond G.P., Fineran P.C. (2014). A widespread bacteriophage abortive infection system functions through a Type IV toxin-antitoxin mechanism. Nucleic Acids Res..

[98] Rye P.T., Delaney J.C., Netirojjanakul C., Sun D.X., Liu J.Z., Essigmann J.M. (2008). Mismatch repair proteins collaborate with methyltransferases in the repair of *O*^6^-methylguanine. DNA Repair.

[99] Ehling-Schulz M., Lereclus D., Koehler T.M. (2019). The *Bacillus cereus* Group: *Bacillus* Species with Pathogenic Potential. Microbiol. Spectr..

[100] Enosi Tuipulotu D., Mathur A., Ngo C., Man S.M. (2021). *Bacillus cereus*: Epidemiology, Virulence Factors, and Host-Pathogen Interactions. Trends Microbiol..

[101] Compant S., Clément C., Sessitsch A. (2010). Plant growth-promoting bacteria in the rhizo- and endosphere of plants: Their role, colonization, mechanisms involved and prospects for utilization. Soil Biol. Biochem..

[102] Fracchia F., Mangeot-Peter L., Jacquot L., Martin F., Veneault-Fourrey C., Deveau A. (2021). Colonization of Naive Roots from *Populus tremula* × *alba* Involves Successive Waves of Fungi and Bacteria with Different Trophic Abilities. Appl. Environ. Microbiol..

[103] Veach A.M., Morris R., Yip D.Z., Yang Z.K., Engle N.L., Cregger M.A., Tschaplinski T.J., Schadt C.W. (2019). Rhizosphere microbiomes diverge among *Populus trichocarpa* plant-host genotypes and chemotypes, but it depends on soil origin. Microbiome.

[104] Pleban S., Chernin L., Chet I. (1997). Chitinolytic activity of an endophytic strain of *Bacillus cereus*. Lett. Appl. Microbiol..

[105] Hu H., Gao Y., Li X., Chen S., Yan S., Tian X. (2020). Identification and Nematicidal Characterization of Proteases Secreted by Endophytic Bacteria *Bacillus cereus* BCM2. Phytopathology.

[106] Sunkar S., Nachiyar C.V. (2012). Biogenesis of antibacterial silver nanoparticles using the endophytic bacterium *Bacillus cereus* isolated from *Garcinia xanthochymus*. Asian Pac. J. Trop. Biomed..

[107] Wang Y., Zhao Q., Sun Z., Li Y., He H., Zhang Y., Yang X., Wang D., Dong B., Zhou H. (2022). Whole-genome analysis revealed the growth-promoting mechanism of endophytic bacterial strain Q2H1 in potato plants. Front. Microbiol..

[108] Rodriguez M., Reina J.C., Sampedro I., Llamas I., Martinez-Checa F. (2022). *Peribacillus castrilensis* sp. nov.: A Plant-Growth-Promoting and Biocontrol Species Isolated From a River Otter in Castril, Granada, Southern Spain. Front. Plant Sci..

[109] Montecillo J.A.V., Bae H. (2022). In Silico analysis of koranimine, a cyclic imine compound from *Peribacillus frigoritolerans* reveals potential nematicidal activity. Sci. Rep..

[110] Gan H.Y., Gan H.M., Savka M.A., Triassi A.J., Wheatley M.S., Smart L.B., Fabio E.S., Hudson A.O. (2014). Whole-genome sequences of 13 endophytic bacteria isolated from shrub willow (*Salix*) grown in geneva, new york. Genome Announc..

[111] Upreti R., Thomas P. (2015). Root-associated bacterial endophytes from *Ralstonia solanacearum* resistant and susceptible tomato cultivars and their pathogen antagonistic effects. Front. Microbiol..

[112] Wang S., Ji B., Su X., Li H., Dong C., Chen S., Zhu Y., Feng W. (2020). Isolation of endophytic bacteria from *Rehmannia glutinosa* Libosch and their potential to promote plant growth. J. Gen. Appl. Microbiol..

[113] Kukla M., Plociniczak T., Piotrowska-Seget Z. (2014). Diversity of endophytic bacteria in *Lolium perenne* and their potential to degrade petroleum hydrocarbons and promote plant growth. Chemosphere.

[114] Husseiny S., Dishisha T., Soliman H.A., Adeleke R., Raslan M. (2021). Characterization of growth promoting bacterial endophytes isolated from *Artemisia annua* L. S. Afr. J. Bot..

[115] Scharschmidt T.C., Fischbach M.A. (2013). What Lives On Our Skin: Ecology, Genomics and Therapeutic Opportunities Of the Skin Microbiome. Drug Discov. Today Dis. Mech..

[116] Thomas P., Swarna G.K., Roy P.K., Patil P. (2008). Identification of culturable and originally non-culturable endophytic bacteria isolated from shoot tip cultures of banana cv. Grand Naine. Plant Cell Tissue Organ Cult..

[117] Brown S.D., Utturkar S.M., Klingeman D.M., Johnson C.M., Martin S.L., Land M.L., Lu T.Y., Schadt C.W., Doktycz M.J., Pelletier D.A. (2012). Twenty-one genome sequences from *Pseudomonas* species and 19 genome sequences from diverse bacteria isolated from the rhizosphere and endosphere of *Populus deltoides*. J. Bacteriol..

[118] Tambong J.T., Xu R., Bromfield E.S.P. (2017). *Pseudomonas canadensis* sp. nov., a biological control agent isolated from a field plot under long-term mineral fertilization. Int. J. Syst. Evol. Microbiol..

[119] Afzal I., Shinwari Z.K., Sikandar S., Shahzad S. (2019). Plant beneficial endophytic bacteria: Mechanisms, diversity, host range and genetic determinants. Microbiol. Res..

