# Supplementary material for: Culturable Bacterial Endophytes of Wild White Poplar (Populus alba L.) Roots: A First Insight into Their Plant Growth-Stimulating and Bioaugmentation Potential
Source: Biology (Basel). 2023 Dec 12;12(12):1519. doi: 10.3390/biology12121519 (PMC10740426; doi:10.3390/biology12121519)
Supplement: Supplementary file 1 [file biology-12-01519-s001.zip › Supplementary Tables.pdf]

Supplementary Table S1. Results of pairwise comparisons of genomes of s4, s23 and closest type strains (TYGS)

Query strain	Subject strain	dDDH (d0, in %)	C.I. (d0, in %)	dDDH (d4, in %)	C.I. (d4, in %)	dDDH (d6, in %)	C.I. (d6, in %)	G+C content difference (in %)
's23'	*Peribacillus frigoritolerans* DSM 8801	87.4	[83.9 - 90.3]	89.3	[86.9 - 91.3]	90.5	[87.8 - 92.6]	0.47
's4'	*Peribacillus castrilensis N3*	79.4	[75.5 - 82.9]	84.5	[81.7 - 86.9]	83.2	[79.9 - 86.1]	0.32
's23'	*Peribacillus castrilensis N3*	77	[73.1 - 80.6]	75.6	[72.6 - 78.4]	79.6	[76.2 - 82.7]	0.1
's4'	's23.fa'	76.2	[72.2 - 79.8]	75.2	[72.2 - 78.0]	78.8	[75.4 - 81.9]	0.43
's4'	*Peribacillus frigoritolerans* DSM 8801	79.5	[75.6 - 83.0]	74.7	[71.7 - 77.5]	81.6	[78.2 - 84.5]	0.04
's23'	*Bacillus cereus* ATCC 14579	13.2	[10.5 - 16.5]	53	[50.3 - 55.6]	13.7	[11.3 - 16.5]	4.88
's4'	Peribacillus simplex NBRC 15720	68.8	[64.9 - 72.4]	52.6	[49.9 - 55.2]	67.1	[63.7 - 70.4]	0.79
's23'	*Peribacillus simplex* NBRC 15720	65.2	[61.4 - 68.9]	51.8	[49.1 - 54.5]	63.9	[60.5 - 67.1]	0.36
's23'	*Bacillus thuringiensis* ATCC 10792	13.1	[10.4 - 16.4]	47.7	[45.1 - 50.3]	13.5	[11.2 - 16.3]	5.34

Supplementary Table S2. Results of pairwise comparisons of genomes of s9 strain and closest type strains (TYGS)

**Query strain**	**Subject strain**	**dDDH (d0, in %)**	**C.I. (d0, in %)**	**dDDH (d4, in %)**	**C.I. (d4, in %)**	**dDDH (d6, in %)**	**C.I. (d6, in %)**	**G+C content difference (in %)**
s9	*Micrococcus aloeverae* BCRC 80870	81.9	[78.0 - 85.2]	83.6	[80.8 - 86.0]	85.1	[82.0 - 87.8]	0.17
s9	*Micrococcus yunnanensis* BCRC 80243	83.5	[79.7 - 86.7]	82.8	[80.0 - 85.3]	86.3	[83.2 - 88.9]	0.23
s9	*Micrococcus yunnanensis* DSM 21948	83.6	[79.7 - 86.8]	82.8	[80.0 - 85.3]	86.4	[83.3 - 89.0]	0.07
s9	*Micrococcus yunnanensis* DSM 24531	81	[77.1 - 84.4]	77.9	[75.0 - 80.7]	83.4	[80.2 - 86.3]	0.21
s9	*Micrococcus luteus* NCTC 2665	77.9	[74.0 - 81.5]	74.8	[71.8 - 77.6]	80.2	[76.8 - 83.2]	0.17
s9	*Micrococcus luteus* ATCC 4698	78.2	[74.2 - 81.7]	74.7	[71.7 - 77.5]	80.4	[77.0 - 83.4]	0.25
s9	*Ottowia caeni* BD-1	12.8	[10.2 - 16.1]	45.3	[42.8 - 47.9]	13.3	[10.9 - 16.0]	9.19
s9	*Micrococcus endophyticus* BCRC 16908	32.4	[29.0 - 36.0]	42.5	[40.0 - 45.1]	32.7	[29.8 - 35.8]	1.58
s9	*Micrococcus endophyticus* DSM 17945	59.4	[55.8 - 63.0]	32.5	[30.1 - 35.0]	52.2	[49.1 - 55.2]	0.74

.

Supplementary Table S3. NCBI Assembly accession numbers of the genomic sequences.

**Strain**	**Taxonomic classification**	**Sample accession**	**Assembly accession**
s2	*Pseudomonas siliginis*	SAMN38245985	JAXDEZ000000000
s3	*Staphylococcus haemolyticus*	SAMN38245986	JAXDEY000000000
s4	*Peribacillus frigoritolerans*	SAMN38245987	JAXDEX000000000
s5	*Corynebacterium amycolatum*	SAMN38245988	JAXDEW000000000
s9	*Micrococcus luteus*	SAMN38245989	JAXDEV000000000
s10	*Pseudomonas canadensis*	SAMN38245990	JAXDEU000000000
s12	*Kocuria rosea*	SAMN38245991	JAXDET000000000
s13	*Bacillus cereus*	SAMN38245992	JAXDES000000000
s14	*Bacillus bombysepticus / B. cereus*	SAMN38245993	JAXDER000000000
s15	*Pseudomonas sp.*	SAMN38245994	JAXDEQ000000000
s17	*Kocuria rosea*	SAMN38245995	JAXDEP000000000
s20	*Pseudomonas sp.*	SAMN38245996	JAXDEO000000000
s22	*Bacillus cereus*	SAMN38245997	JAXDEN000000000
s23	*Peribacillus frigoritolerans*	SAMN38245998	JAXDEM000000000
