# Supplementary material for: Culturable Bacterial Endophytes of Wild White Poplar (Populus alba L.) Roots: A First Insight into Their Plant Growth-Stimulating and Bioaugmentation Potential
Source: Biology (Basel). 2023 Dec 12;12(12):1519. doi: 10.3390/biology12121519 (PMC10740426; doi:10.3390/biology12121519)
Supplement: Supplementary file 1 [file biology-12-01519-s001.zip › Supplementary_Figures.pdf]

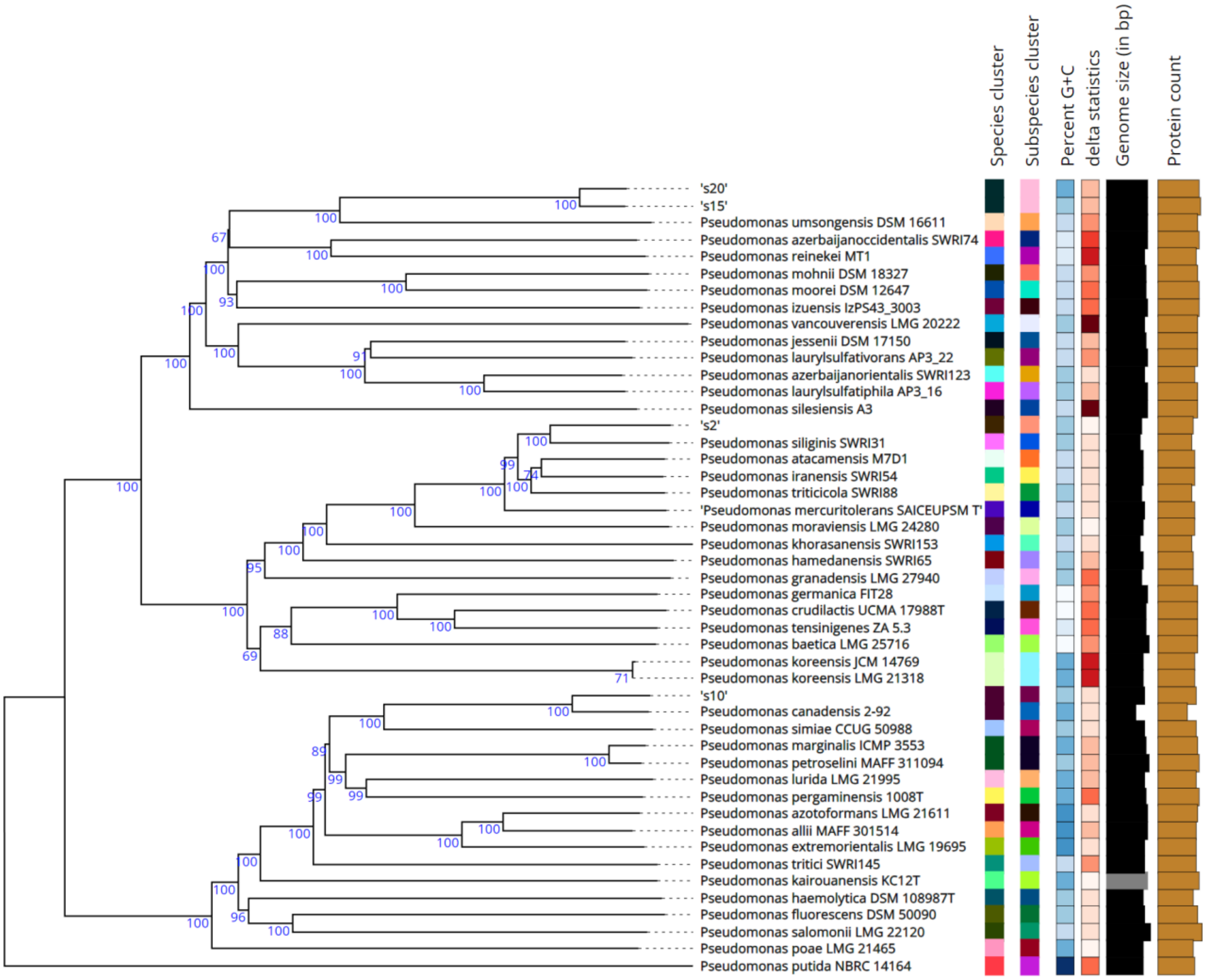

Supplementary Figure S1. Phylogenetic tree inferred by TYGS for type strains with highest genomic similarity scores (MASH algorithm) to s2, s10, s15, s20 strains. *P. putida*, *P. fluorescens* are added manually.

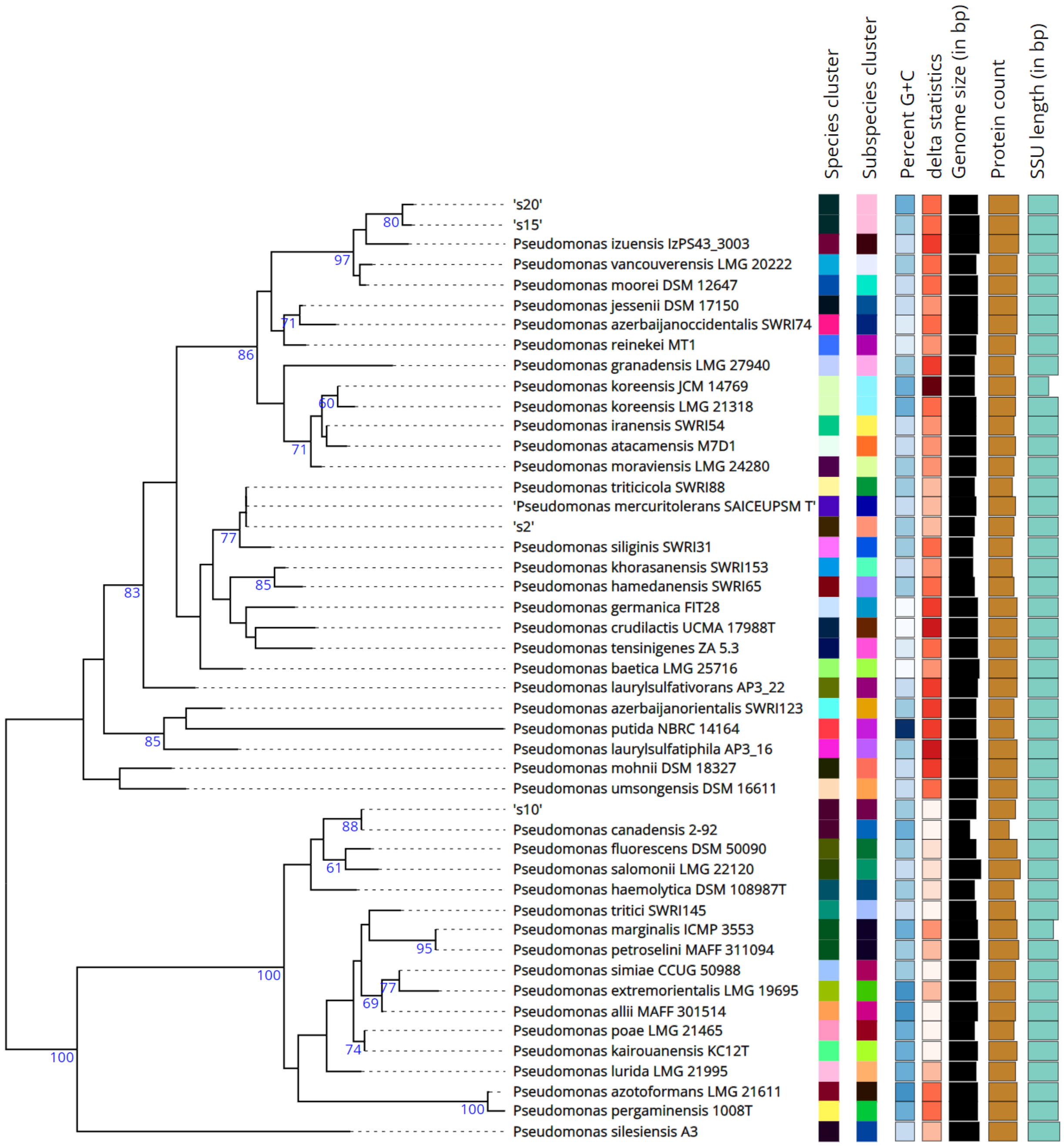

Supplementary Figure S2. Phylogenetic tree inferred by TYGS for type strains with highest 16S similarity scores to s2, s10, s15, s20 strains. *P. putida*, *P. fluorescens* are added manually.

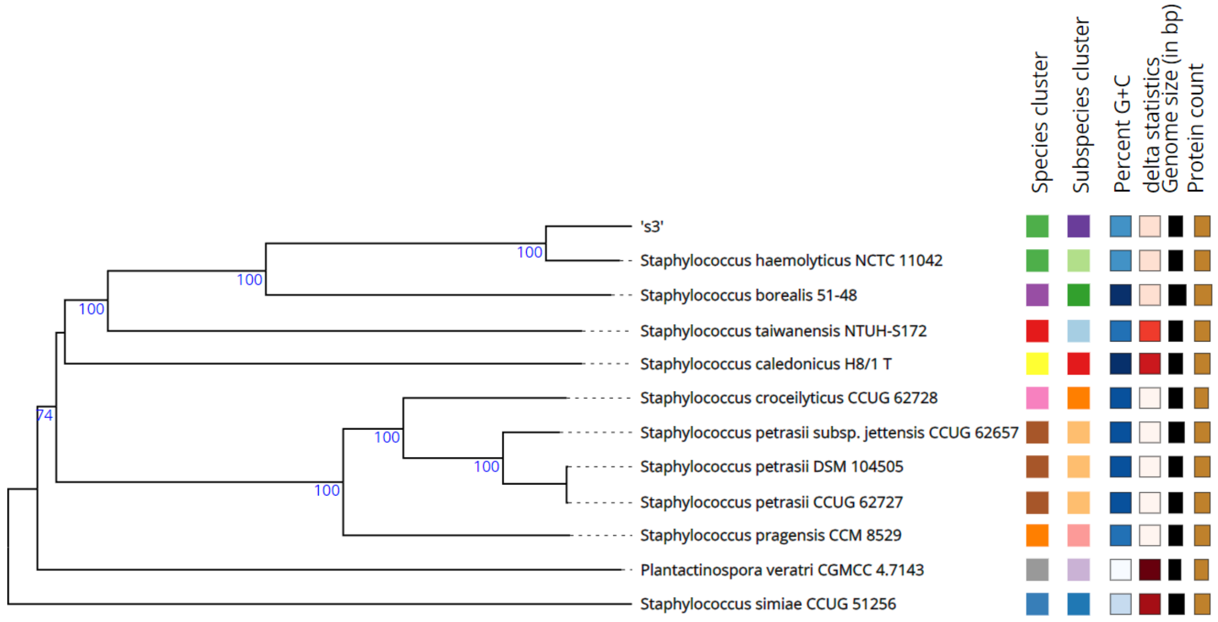

Supplementary Figure S3. Phylogenetic tree inferred by TYGS for type strains with highest genomic similarity scores (MASH algorithm) to s3 strain.

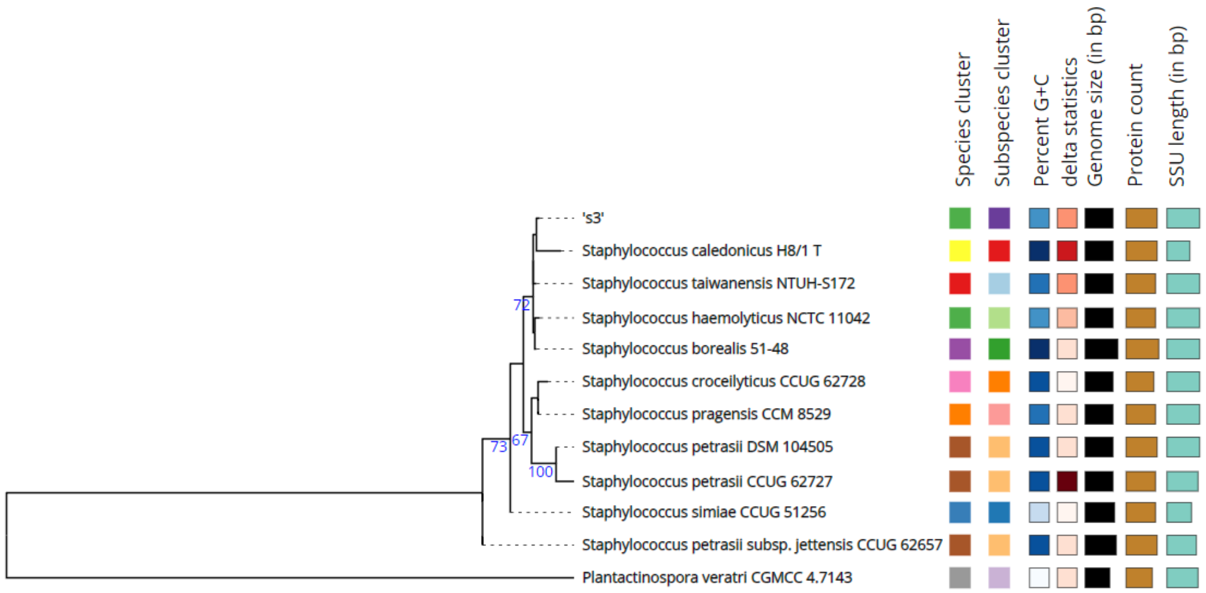

Supplementary Figure S4. Phylogenetic tree inferred by TYGS for type strains with highest 16S similarity scores to s3 strain.

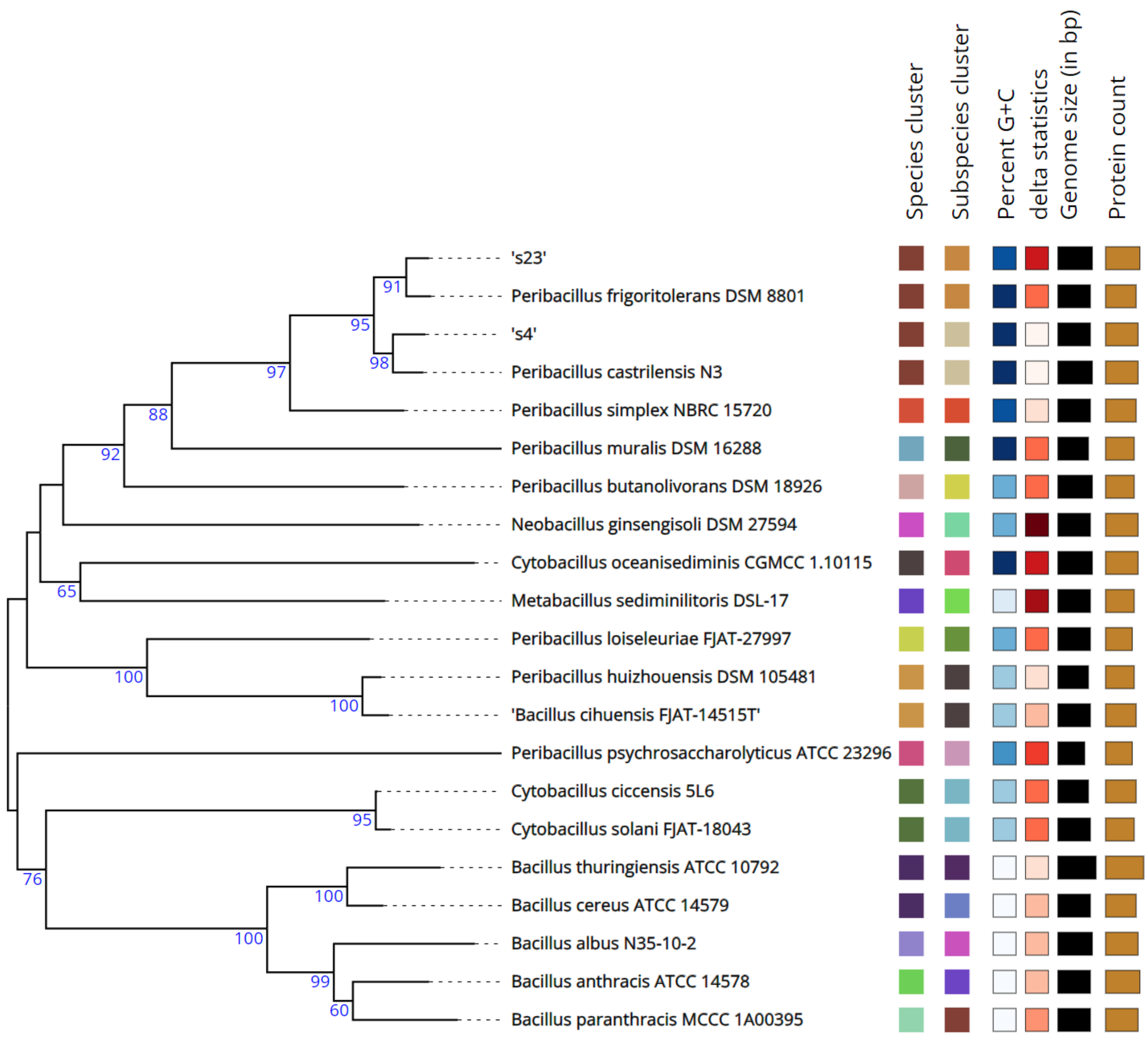

Supplementary Figure S5. Phylogenetic tree inferred by TYGS for type strains with highest genomic similarity scores (MASH algorithm) to s4, s23 strains.

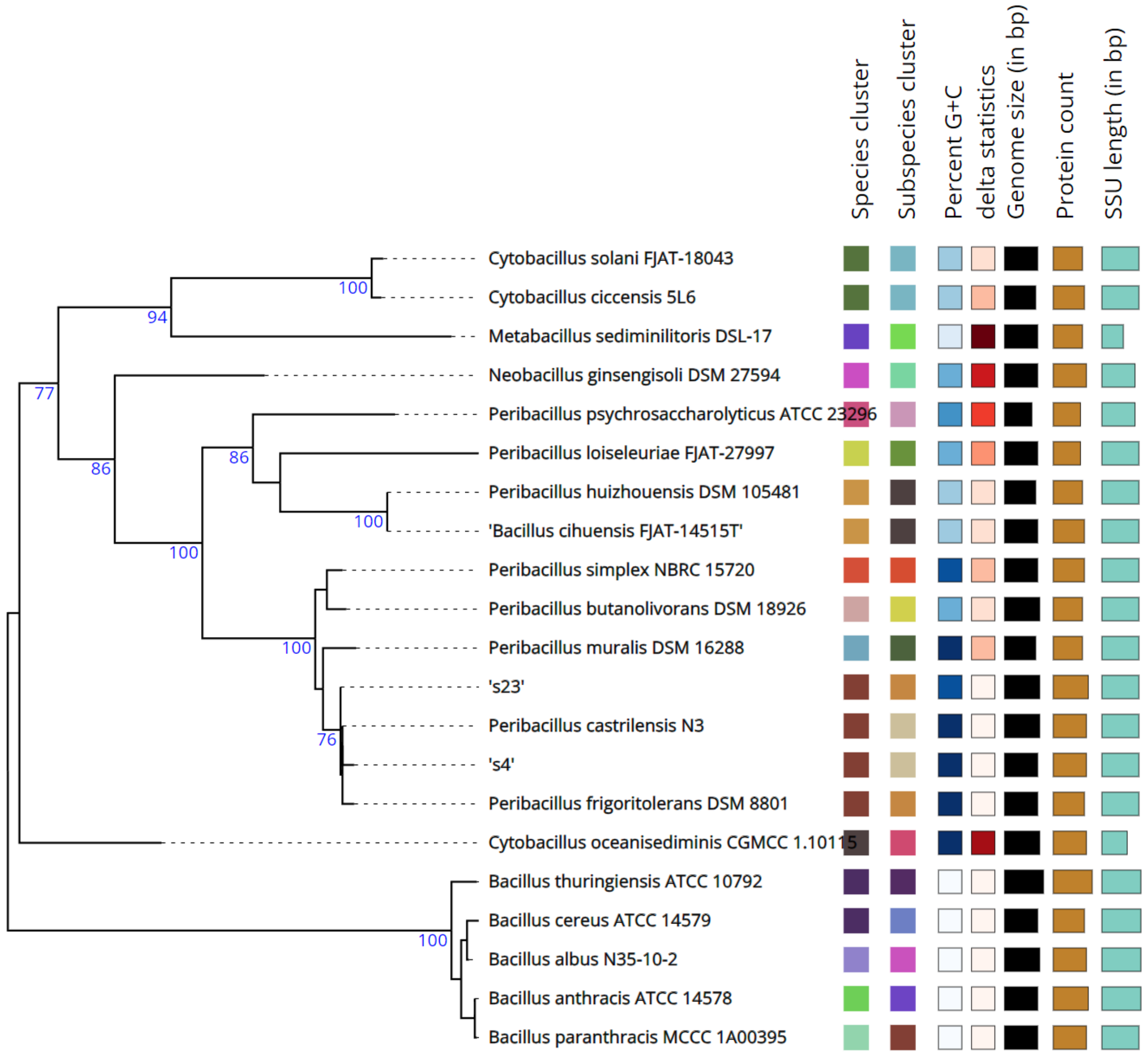

Supplementary Figure S6. Phylogenetic tree inferred by TYGS for type strains with highest 16S similarity scores to s4, s23 strains.

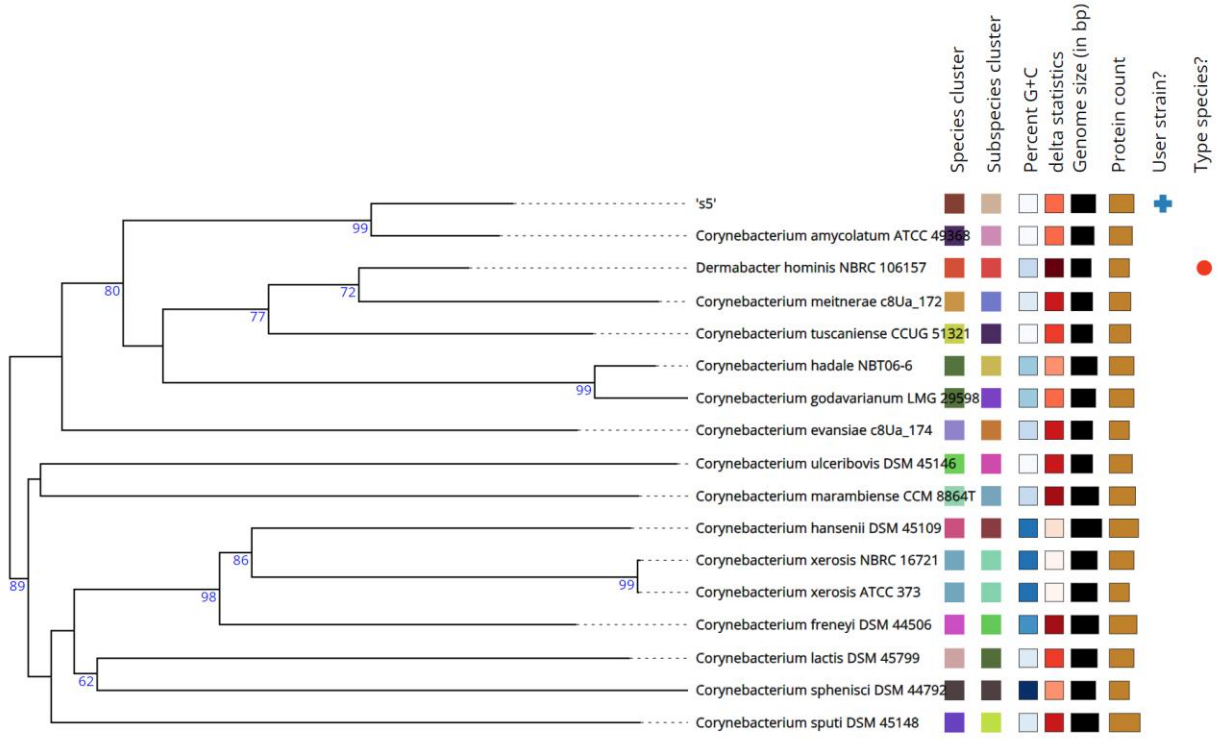

Supplementary Figure S7. Phylogenetic tree inferred by TYGS for type strains with highest genomic similarity scores (MASH algorithm) to s5 strain.

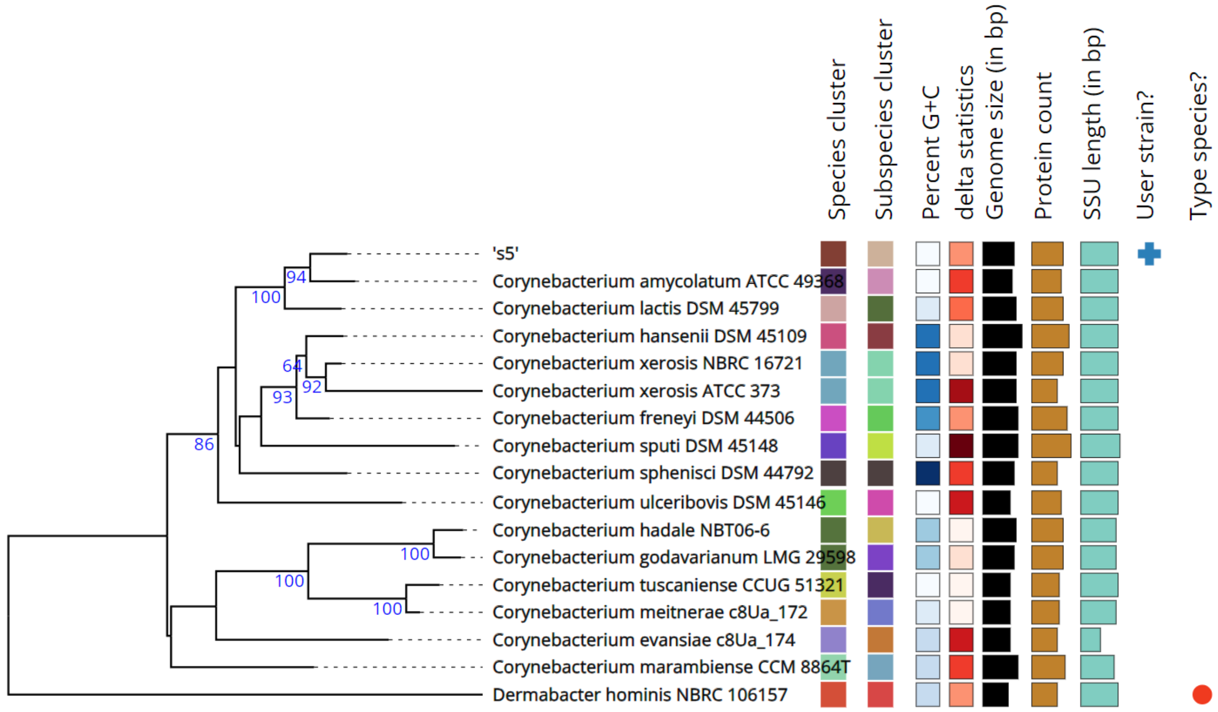

Supplementary Figure S8. Phylogenetic tree inferred by TYGS for type strains with highest 16S similarity scores to s5 strain.

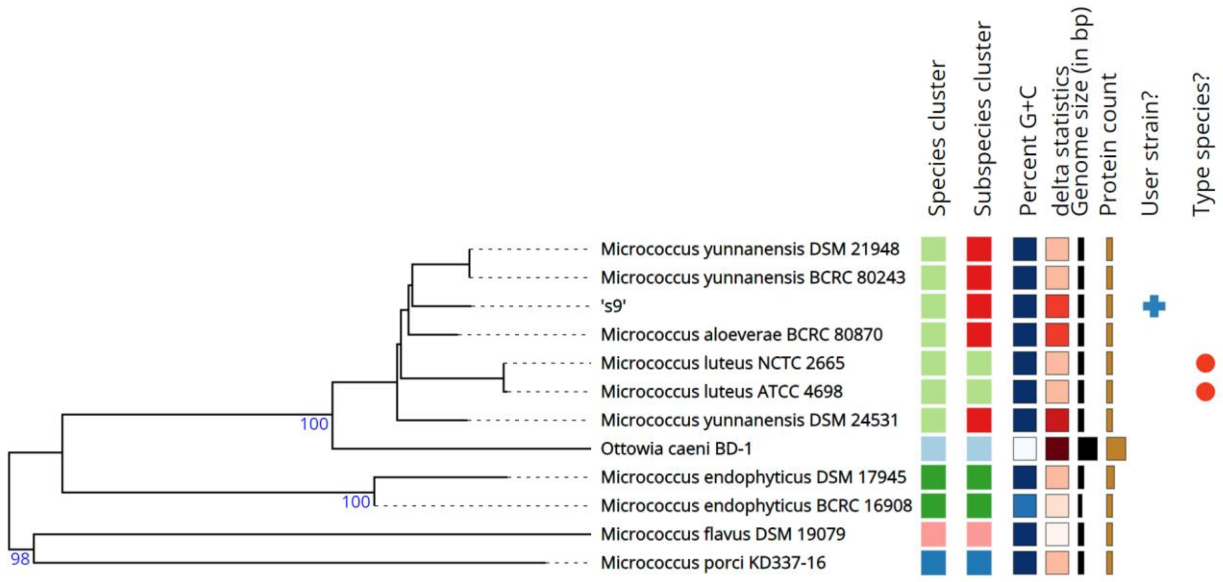

Supplementary Figure S9. Phylogenetic tree inferred by TYGS for type strains with highest genomic similarity scores (MASH algorithm) to s9 strain.

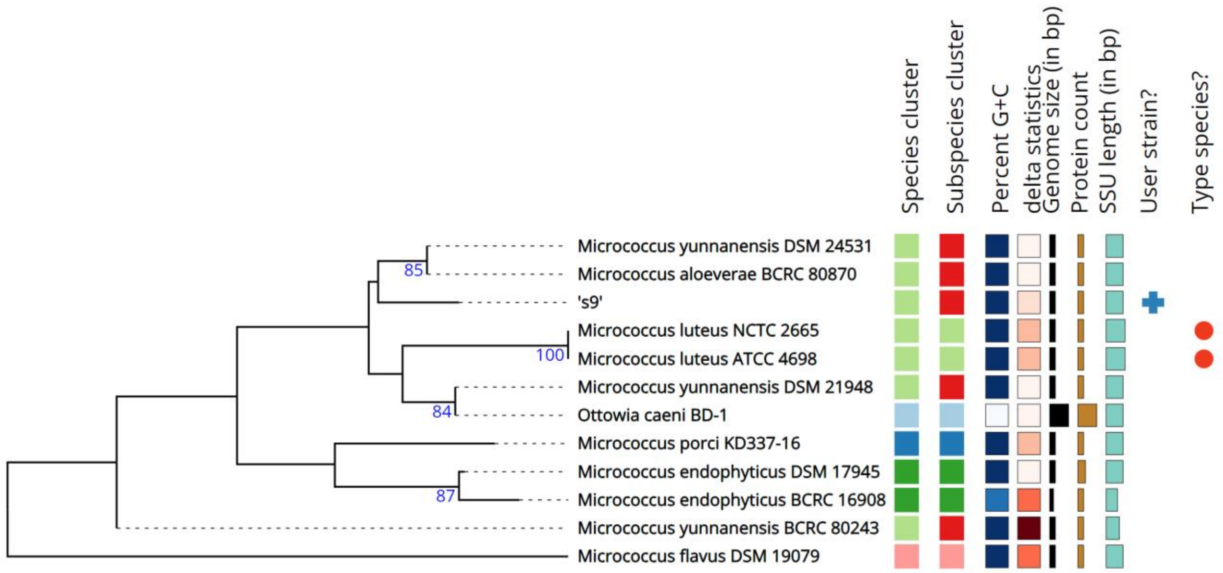

Supplementary Figure S10. Phylogenetic tree inferred by TYGS for type strains with highest 16S similarity scores to s9 strain.

.

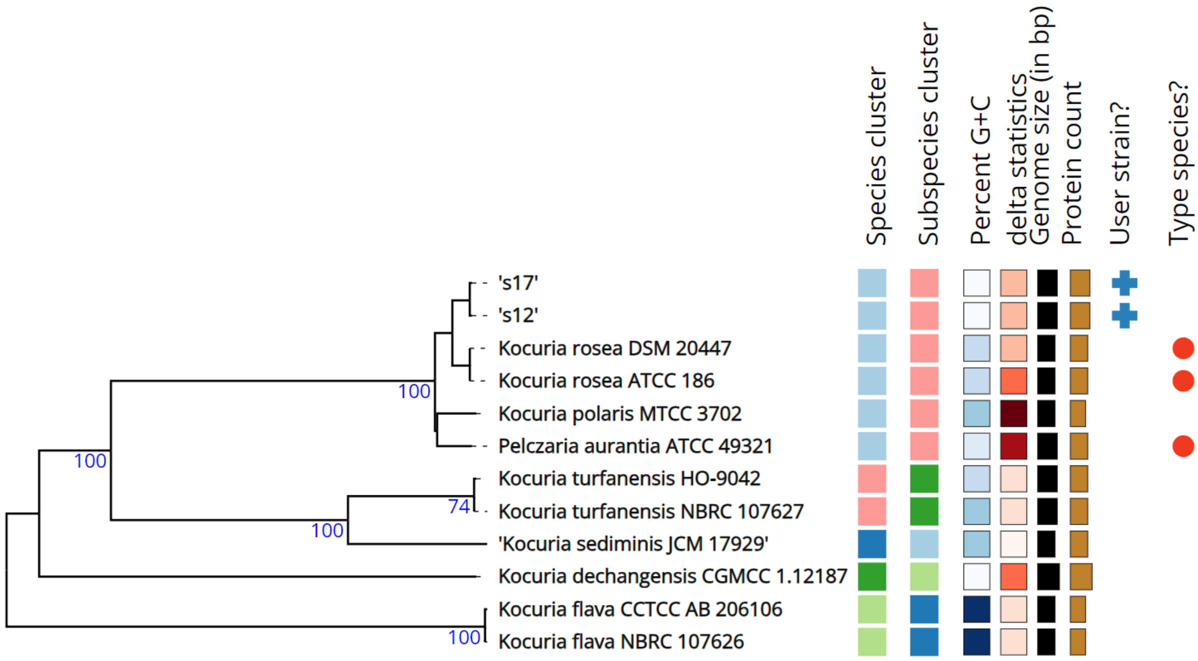

Supplementary Figure S11. Phylogenetic tree inferred by TYGS for type strains with highest genomic similarity scores (MASH algorithm) to s12 and s17 strains.

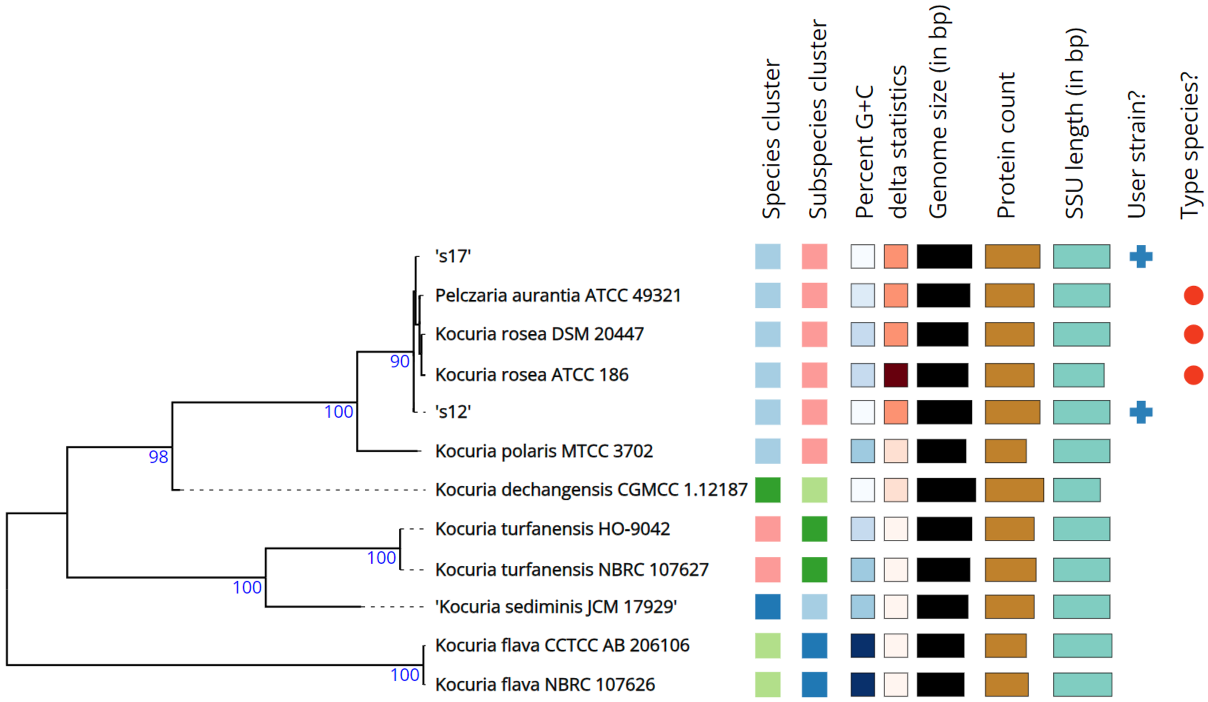

Supplementary Figure S12. Phylogenetic tree inferred by TYGS for type strains with highest 16S similarity scores to s12 and s17 strains.

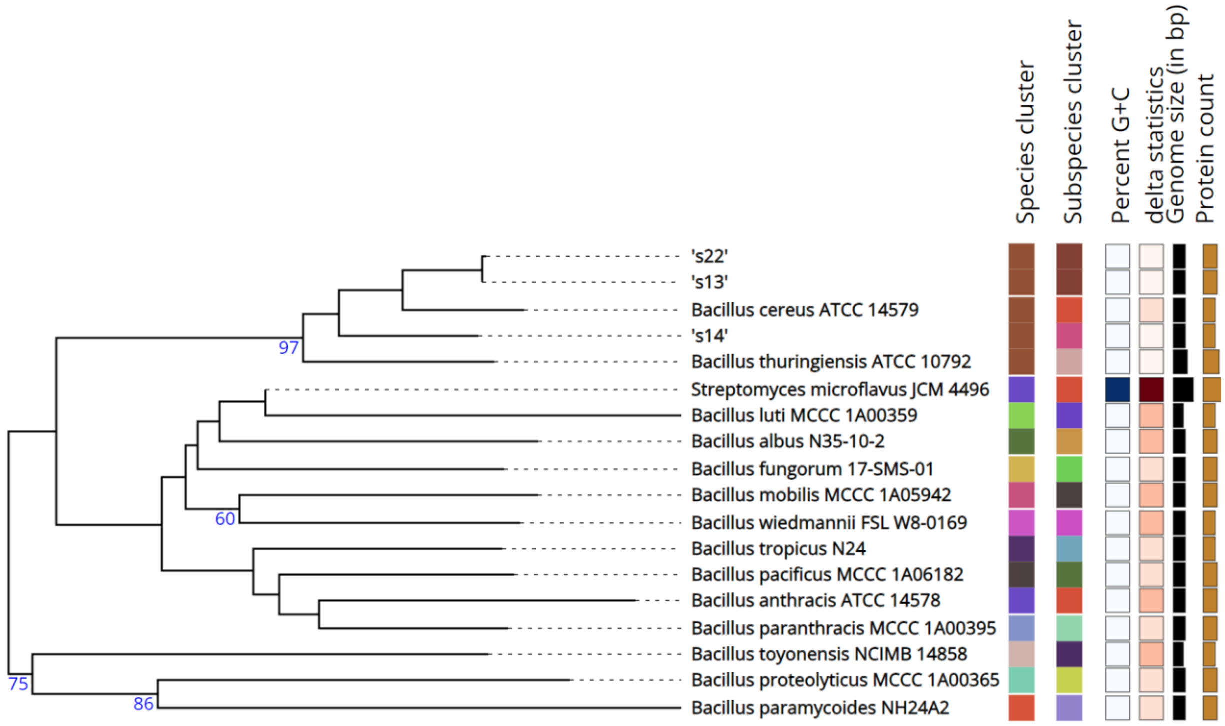

Supplementary Figure S13. Phylogenetic tree inferred by TYGS for type strains with highest genomic similarity scores (MASH algorithm) to s13, s14 and s22 strains.

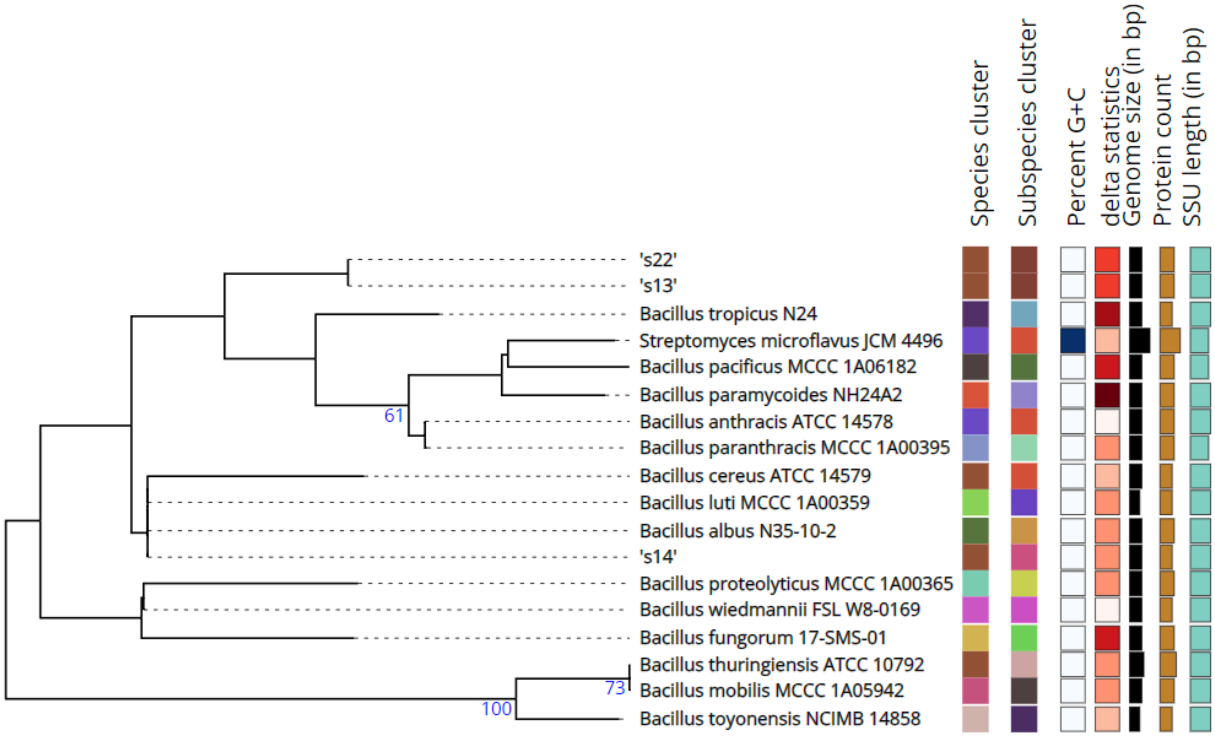

Supplementary Figure S14. Phylogenetic tree inferred by TYGS for type strains with highest 16S similarity scores to s13, s14 and s22 strains.
